# Prevention of Pertussis, Tetanus, and Diphtheria with Vaccines in the United States: Recommendations of the Advisory Committee on Immunization Practices (ACIP)

**DOI:** 10.15585/mmwr.rr6702a1

**Published:** 2018-04-27

**Authors:** Jennifer L. Liang, Tejpratap Tiwari, Pedro Moro, Nancy E. Messonnier, Arthur Reingold, Mark Sawyer, Thomas A. Clark

**Affiliations:** 1 Division of Bacterial Diseases, National Center for Immunization and Respiratory Diseases, CDC; 2 Division of Healthcare Quality Promotion, National Center for Emerging and Zoonotic Infectious Diseases, CDC; 3 Office of the Director, National Center for Immunization and Respiratory Diseases, CDC; 4 University of California, Berkeley; Berkeley, CA; 5 University of California, San Diego; La Jolla, California; 6 Division of Reproductive Health, National Center for Chronic Disease Prevention and Health Promotion, CDC

## Abstract

This report compiles and summarizes all recommendations from CDC's Advisory Committee on Immunization Practices (ACIP) regarding prevention and control of tetanus, diphtheria, and pertussis in the United States. As a comprehensive summary of previously published recommendations, this report does not contain any new recommendations and replaces all previously published reports and policy notes; it is intended for use by clinicians and public health providers as a resource. ACIP recommends routine vaccination for tetanus, diphtheria, and pertussis. Infants and young children are recommended to receive a 5-dose series of diphtheria and tetanus toxoids and acellular pertussis (DTaP) vaccines, with one adolescent booster dose of tetanus toxoid, reduced diphtheria toxoid, and acellular pertussis (Tdap) vaccine. Adults who have never received Tdap also are recommended to receive a booster dose of Tdap. Women are recommended to receive a dose of Tdap during each pregnancy, which should be administered from 27 through 36 weeks’ gestation, regardless of previous receipt of Tdap. After receipt of Tdap, adolescents and adults are recommended to receive a booster tetanus and diphtheria toxoids (Td) vaccine every 10 years to assure ongoing protection against tetanus and diphtheria.

## Introduction

This report compiles and summarizes all previously published recommendations from CDC’s Advisory Committee on Immunization Practices (ACIP) regarding prevention and control of pertussis, tetanus, and diphtheria in the United States, specifically after the introduction of acellular pertussis vaccines, and does not contain any new recommendations. A timeline of ACIP recommendations for DTaP and Tdap during 1991–2015 is available at https://stacks.cdc.gov/view/cdc/52821. This report describes the process undertaken and the rationale used in support of these recommendations and is intended for use by clinicians and public health providers as a resource.

From the late 1940s through the 1990s, vaccination against pertussis, diphtheria, and tetanus with a combined diphtheria and tetanus toxoids and whole-cell pertussis (DTP) vaccine was recommended for infants and young children. Receipt of DTP was commonly associated with local adverse events (e.g., redness, swelling, and pain at the injection site) and less commonly with serious adverse events (*1*,*2*). Because of safety concerns about the whole-cell pertussis component of DTP, diphtheria and tetanus toxoids and acellular pertussis (DTaP) vaccines were developed and subsequently replaced doses of DTP in the 1990s. Since 1997, infants and young children have been recommended to receive a 5-dose series of DTaP (*3*). In 2005, ACIP recommended that adolescents and adults receive a single dose of a tetanus toxoid, reduced diphtheria toxoid, and acellular pertussis (Tdap) vaccine (*4*,*5*). After receipt of Tdap, adolescents and adults are recommended to receive a booster dose of tetanus and diphtheria toxoids (Td) vaccine every 10 years or when indicated for wound management (*4*,*5*). In 2012, in an effort to reduce the burden of pertussis in infants, ACIP recommended a dose of Tdap for women during each pregnancy (*6*).

For the purposes of this report, DTaP and Tdap are used as general terms for diphtheria toxoid, tetanus toxoid, and acellular pertussis vaccines, and DT and Td are used for diphtheria and tetanus toxoid–containing vaccines. Any of the vaccine formulations licensed in the United States can be used in an age-appropriate manner to implement these vaccination recommendations. Both DTP and monovalent tetanus toxoid (TT) vaccines are discussed for historical purposes and no longer are manufactured or available in the United States.

ACIP recommendations for vaccination for pertussis, tetanus, and diphtheria and guidance for use are described elsewhere in this report (see Recommendations for Use of Pertussis, Tetanus, and Diphtheria Vaccines) (Table 1). Details regarding contraindications, precautions, and special circumstances are described elsewhere in this report (see Recommendations for Use of Pertussis, Tetanus, and Diphtheria Vaccines) (Tables 2 and 3). In 2013, after review of available data, ACIP did not support a universal recommendation for a second dose of Tdap for the general population (see No Additional Doses of Tdap For the General Population). In 2014 and 2015, ACIP did not support a second dose of Tdap for health care personnel or close contacts of infants.

**TABLE 1:** Recommended pertussis, diphtheria, and tetanus vaccination schedule — Advisory Committee on Immunization Practices, 2017

Vaccine	Age group/Indication	Recommended schedule
DTaP*	2 mos–6 yrs	Primary (3 doses) • 1 dose at ages 2, 4, and 6 mos 1st booster • 1 dose at age 15–18 mos 2nd booster • 1 dose at age 4–6 yrs
Tdap^†^	7–10 yrs^§^	Not routinely recommended; refer to “Persons with incomplete or unknown vaccine history”
	11–18 yrs	11–12 yrs, 1 dose 13–18 yrs, 1 dose if not vaccinated previously with Tdap
	≥19 yrs	1 dose if not vaccinated previously with Tdap
	Pregnant women^¶^	1 dose each pregnancy; preferred at 27–36 wks’ gestation
Td^†^		Booster • 1 dose every 10 yrs

**Abbreviations**: DTaP = diphtheria and tetanus toxoids and acellular pertussis vaccine; Td = tetanus and diphtheria toxoids vaccine; Tdap = tetanus toxoid, reduced diphtheria toxoid, and acellular pertussis vaccine.

*See Table 4 for vaccine type and ages for licensed use.

^†^ See Table 5 for vaccine type and ages for licensed use.

^§^ Off-label use of Tdap in persons aged 7–9 years.

^¶^ Off-label use of Tdap.

**TABLE 2:** Contraindications and precautions* for DTaP, Tdap, DT, and Td vaccines

Vaccine	Contraindications	Precautions*
DTaP	Severe allergic reaction (e.g., anaphylaxis) after a previous dose or to a vaccine component^†,§^ Encephalopathy (e.g., coma, decreased level of consciousness, or prolonged seizures) not attributable to another identifiable cause within 7 days of administration of previous dose of DTP or DTaP^¶^	Progressive or unstable neurologic disorder, including infantile spasms, uncontrolled seizures or progressive encephalopathy; defer DTaP until neurologic status clarified and stabilized Guillain-Barré syndrome <6 weeks after previous dose of tetanus toxoid–containing vaccine History of Arthus-type hypersensitivity reactions after a previous dose of tetanus or diphtheria toxoid–containing vaccines; defer vaccination until at least 10 years have e–lapsed since the last tetanus toxoid–containing vaccine Moderate or severe acute illness with or without fever
Tdap	Severe allergic reaction (e.g., anaphylaxis) after a previous dose or to a vaccine component^§^ Encephalopathy (e.g., coma, decreased level of consciousness, or prolonged seizures) not attributable to another identifiable cause, within 7 days of administration of previous dose of DTP, DTaP, or Tdap**	Progressive or unstable neurological disorder, uncontrolled seizures, or progressive encephalopathy until a treatment regimen has been established and the condition has stabilized; these precautions are for pertussis components Guillain-Barré syndrome <6 weeks after a previous dose of tetanus toxoid–containing vaccine History of Arthus-type hypersensitivity reactions after a previous dose of tetanus or diphtheria toxoid–containing vaccines; defer vaccination until at least 10 years have elapsed since the last tetanus toxoid–containing vaccine Moderate or severe acute illness with or without fever
DT, Td	Severe allergic reaction (e.g., anaphylaxis) after a previous dose or to a vaccine component^§^	Guillain-Barré syndrome <6 weeks after previous dose of tetanus toxoid–containing vaccine History of Arthus-type hypersensitivity reactions after a previous dose of tetanus or diphtheria toxoid–containing vaccines; defer vaccination until at least 10 years have elapsed since the last tetanus toxoid–containing vaccine Moderate or severe acute illness with or without fever

**Abbreviations**: DT = diphtheria and tetanus toxoids vaccine; DTaP = diphtheria and tetanus toxoids and acellular pertussis vaccine; DTP = diphtheria toxoid, tetanus toxoid and whole-cell pertussis vaccine; MenACWY = quadrivalent meningococcal conjugate, serogroups A, C, W, Y vaccine; Td = tetanus and diphtheria toxoids vaccine; Tdap = tetanus toxoid, reduced diphtheria toxoid, and acellular pertussis vaccine.

* Events or conditions listed as precautions should be reviewed carefully. Benefits of and risks for administering a specific vaccine to a person under these circumstances should be considered. If the risk from the vaccine is believed to outweigh the benefit, the vaccine should not be administered. If the benefit of vaccination is believed to outweigh the risk, the vaccine should be administered. Whether and when to administer DTaP to children with proven or suspected underlying neurologic disorders should be decided on a case-by-case basis.

^†^ Further vaccination with any of the three components of DTaP should be deferred because of uncertainty as to which component of the vaccine might be responsible.

^§^ Because of the importance of tetanus vaccination, persons who experience anaphylactic reactions should be referred to an allergist to determine whether they have a specific allergy to tetanus toxoid and can be desensitized to tetanus toxoid.

^¶^ In such cases, DT vaccine should be administered for the remaining doses in the vaccination schedule to ensure protection against diphtheria and tetanus.

** This contraindication is for the pertussis component, and these persons should receive Td instead of Tdap.

**TABLE 3:** Conditions that are not contraindications to vaccination with DTaP, DT, Td, and Tdap

Vaccine	Conditions commonly misperceived as contraindications (i.e., vaccine may be administered under these conditions)
General for DTaP, DT, Td, Tdap	Mild acute illness with or without fever Mild-to-moderate local reaction (i.e., swelling, redness, soreness); low-grade or moderate fever after previous dose Lack of previous physical examination in well-appearing person Current antimicrobial therapy Convalescent phase of illness Preterm birth Recent exposure to an infectious disease History of penicillin allergy, other nonvaccine allergies, relatives with allergies, or receiving allergen extract immunotherapy
DTaP	Fever of <105°F (<40.5°C), fussiness or mild drowsiness after a previous dose of DTP/DTaP Family history of seizures Family history of sudden infant death syndrome Family history of an adverse event after DTP or DTaP administration Stable neurologic conditions (e.g., cerebral palsy, well-controlled seizures, or developmental delay) History of collapse or shock-like state (i.e., hypotonic hyporesponsive episode) within 48 hours after receiving a previous dose of DTP/DTaP History of seizure with or without fever within 3 days after receiving a previous dose of DTP/DTaP History of persistent, inconsolable crying lasting >3 hours within 48 hours after receiving a previous dose of DTP/DTaP
Tdap	Fever of ≥105°F (≥40.5°C) for <48 hours after vaccination with a previous dose of DTP or DTaP History of collapse or shock-like state (i.e., hypotonic hyporesponsive episode) within 48 hours after receiving a previous dose of DTP/DTaP History of seizure with or without fever within 3 days after receiving a previous dose of DTP/DTaP History of persistent, inconsolable crying lasting >3 hours within 48 hours after receiving a previous dose of DTP/DTaP History of extensive limb swelling after DTP/DTaP/Td that is not an Arthus-type reaction Stable neurologic disorder History of brachial neuritis Breastfeeding Immunosuppression

**Abbreviations**: DT = diphtheria and tetanus toxoids vaccine; DTaP = diphtheria and tetanus toxoids and acellular pertussis vaccine; DTP = diphtheria toxoid, tetanus toxoid and whole-cell pertussis vaccine; Td = tetanus and diphtheria toxoids vaccine; Tdap = tetanus toxoid, reduced diphtheria toxoid, and acellular pertussis vaccine.

**Source:** Adapted from CDC. General recommendations on immunization: recommendations of the Advisory Committee on Immunization Practices (ACIP). MMWR Recomm Rep 2011;60(No. RR-2).

## Methods

Periodically, ACIP reviews available information to inform the development or revision of its vaccine recommendations. In February 2009, the ACIP Pertussis Vaccines Work Group was formed to review and revise previously published vaccine recommendations for DTaP, DT, Td, TT, and Tdap because of 1) the availability of new licensed DTaP vaccine products since 1997; 2) multiple ACIP updates to the adolescent and adult Tdap recommendations; 3) new U.S. Food and Drug Administration (FDA) age indications for both Tdap vaccine products; 4) the need to incorporate pertussis, tetanus, and diphtheria vaccine recommendations into a single document; 5) new data on Tdap coverage, impact, and vaccine effectiveness; and 6) the discontinuation of TT vaccine manufacturing and availability in the United States. The work group held teleconference meetings monthly from April 2009 through April 2015. In addition to ACIP members, the work group included participants from the American Academy of Family Physicians (AAFP), American Academy of Pediatrics (AAP), American College of Obstetricians and Gynecologists (ACOG), the Association of Immunization Managers, CDC, the Council of State and Territorial Epidemiologists, FDA, the Infectious Diseases Society of America, the National Institute of Health, and other infectious disease experts (*7*).*

Issues reviewed and considered by the work group included epidemiology of pertussis, tetanus, and diphtheria in the United States; use of Tdap vaccine among persons aged ≥65 years, children aged 7–10 years, health care personnel, and women during pregnancy; minimum interval between the last tetanus toxoid–containing vaccine and receipt of Tdap; effectiveness of Tdap vaccine; and vaccine safety. Recommendation options were developed and discussed by the work group. The work group evaluated the available published and unpublished data and evidence regarding pertussis disease epidemiology in the United States, decision analyses, cost-effectiveness, programmatic considerations, vaccine immunogenicity, vaccine safety, and postlicensure Tdap vaccine effectiveness. When evidence was lacking, the recommendations incorporated expert opinion of the work group members (*6**,**8*–*10*).

From June 2010 through June 2015 at 11 ACIP meetings, a summary of the data reviewed, work group discussions, and proposed changes to recommendations were presented. During these 11 meetings, changes to recommendations, if made, were approved either as submitted or as amended by ACIP and then published as policy notes in *MMWR*. A summary of these recommendations is available at https://stacks.cdc.gov/view/cdc/52821. During the preparation of this summary report, nonsystematic literature searches for specific topics were conducted in PubMed and Google Scholar for published literature in English available in print or online to provide more updated data and information since publication of any ACIP vaccine recommendations for DTaP, DT, Td, TT, and Tdap published in *MMWR*; a document containing the literature search topics, search terms, search period, and references selected is available at https://stacks.cdc.gov/view/cdc/52823. The contents of this summary report were presented to ACIP and approved at the October 2016 ACIP meeting. During the review process, CDC modified the summary to update and clarify wording. ACIP meeting minutes, including declaration of ACIP member conflicts of interest, are available at https://www.cdc.gov/ vaccines/acip/meetings/meetings-info.html. One ACIP member abstained from voting because of a conflict of interest.

## Background and Epidemiology of Pertussis

Pertussis is an acute respiratory disease caused by the bacterium *Bordetella pertussis* (*11*). Classic pertussis disease is characterized by three phases of illness: catarrhal, paroxysmal, and convalescent (*11*–*13*). During the catarrhal phase, infected persons experience coryza (inflammation of the mucous membranes of the nasal cavities), mild occasional cough, and low-grade fever. The paroxysmal phase is characterized by spasmodic cough, posttussive vomiting, and inspiratory whoop. Symptoms slowly improve during the convalescent phase, which generally lasts 7–10 days, but can last for months. Factors that affect the clinical presentation of pertussis include age, the level of immunity, history of vaccination, and use of antimicrobials early during the course of the illness (*11*).

*B. pertussis* is transmitted primarily from person to person through aerosolized respiratory droplets generated by coughing or sneezing. Persons with pertussis are most infectious during the catarrhal and early paroxysmal phases of illness (*12*). Pertussis generally is treated with antibiotics, which are used to control the signs and symptoms and to prevent infected persons from spreading the infection to others. Recommended antibiotics for pertussis include azithromycin, clarithroymycin, erythromycin, or trimethoprim-sulfamethoxazole (TMP-SMX). Guidance for the treatment of pertussis has been published previously (*14*). Guidance on postexposure prophylaxis of pertussis is available at https://www.cdc.gov/pertussis/outbreaks/pep.html.

### Epidemiology of Pertussis in the United States

**FIGURE 1:**
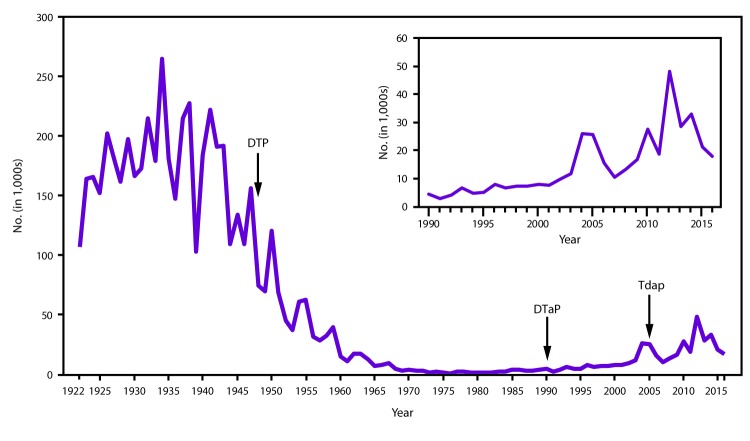
Number of reported pertussis cases — United States, 1922–2016 **Abbreviations:** DTaP = diphtheria and tetanus toxoids and acellular pertussis vaccine; DTP = diphtheria toxoid, tetanus toxoid and whole-cell pertussis vaccine; Tdap = tetanus toxoid, reduced diphtheria toxoid and acellular pertussis vaccine. **Sources:** National Notifiable Diseases Surveillance System and Supplemental Pertussis Surveillance System and 1922–1949, passive reports to the U.S. Public Health Service.

**FIGURE 2:**
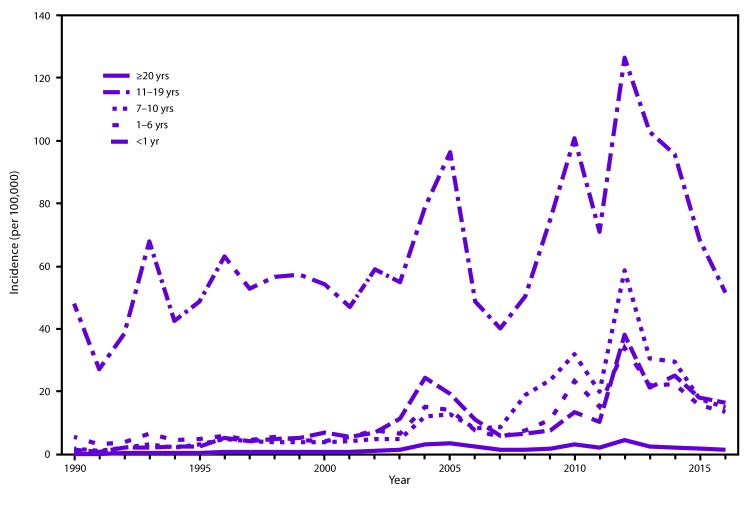
Annual incidence* of pertussis, by age group — United States, 1990–2016 **Sources:** National Notifiable Diseases Surveillance System and Supplemental Pertussis Surveillance System. * Per 100,000 population.

In the United States, pertussis is a nationally notifiable disease (*15*). During 1934–1943, before the introduction of childhood pertussis vaccination in the United States, an annual average of 200,752 pertussis cases and 4,034 pertussis-related deaths were reported (*16*). After introduction of whole-cell pertussis vaccine during the 1940s, the number of reported pertussis cases declined dramatically, reaching an historic low of 1,010 in 1976 (Figure 1) (*3*). Since the 1980s, there has been an overall trend of an increase in reported pertussis cases, especially among adolescents and adults (*17*–*19*). Although pertussis is cyclic in nature, with peaks in disease every 3 to 5 years, the peaks have gotten higher, notably in 2004 (25,827 cases), 2005 (25,616 cases), 2010 (27,500 cases), and 2012 (48,277 cases) (*20*–*24*). The increase in reported pertussis cases is likely attributable to an actual increase in the burden of disease; however, other factors have contributed to the estimates of disease burden. These include improvements in diagnostic testing, increased health care personnel and public awareness of pertussis, and better reporting of cases (*17*,*18*,*25*–*27*). A growing body of evidence strongly suggests that the change in vaccines in the late 1990s from whole-cell pertussis vaccines to acellular pertussis vaccines in the childhood vaccine series has caused the age-specific increases in pertussis incidence among children aged 7–10 years observed in the mid-2000s because of waning immunity (Figure 2) (*28*–*30*).

In the United States, coverage with pertussis-containing vaccines varies across age groups. Vaccination coverage with DTaP in children aged 19–35 months remains consistently high, at 95.0% for ≥3 DTaP doses and 84.6% for ≥4 DTaP doses reported in 2015 (*31*). Coverage for ≥4 DTaP doses is below the *Healthy People 2020* target of 90% (*32*). Since the introduction of Tdap in 2005, coverage with Tdap in adolescents aged 13–17 years has increased substantially, from 10.8% in 2006 to 86.4% in 2015 (*33*,*34*). Tdap coverage among adolescents has met the *Healthy People 2020* target of 80% (*35*). In 2015, for adults aged ≥19 years, the proportion receiving any tetanus toxoid–containing vaccine (e.g, TT, Td, or Tdap) during the preceding 10 years was 62.1% (19–49 years), 64.1% (50–64 years), and 56.9% (≥65 years); Tdap coverage was 23.1% (*36*). Among pregnant women, Tdap coverage during the 2015–2016 influenza season was 48.8% (*37*).

Although pertussis vaccination has resulted in a markedly reduced incidence of pertussis cases and deaths, pertussis still causes morbidity in persons of all ages. Compared with all other age groups, infants aged <12 months have substantially higher rates of pertussis disease, complications, hospitalizations, and pertussis-related deaths (*38*–*40*). The highest percentage of pertussis-related hospitalizations and deaths occurs among infants aged <2 months (CDC, unpublished data, 2016) (*38*,*41*). During 2004–2016, among all infants hospitalized for pertussis, 54.4% were aged <2 months; of the infant pertussis cases who died, 85.5% were aged <2 months and too young to have received any doses of pertussis vaccines (CDC, unpublished data, 2016). Over the past decade, with the changing pertussis epidemiology, a shift in the source of pertussis transmission to infants has been observed, with siblings rather than mothers being the most common source of pertussis infection for infants (*40*,*42*,*43*).

Although infants have substantially higher rates of reported pertussis compared with other age groups, an increase in the number of reported pertussis cases among children and adolescents since the mid-2000s has been attributed to the waning of acellular pertussis vaccine-induced immunity (Figure 2). This increase was first observed in children aged 7–10 years who were among the first birth cohorts to exclusively receive 5 doses of acellular pertussis (DTaP) vaccine following the switch from DTP in 1997 (*28*). As this birth cohort aged, an increase in reported pertussis cases also was observed among those aged 13–14 years in 2012 (*29*,*44*). Continued monitoring of national surveillance data will permit further characterization of the impact of acellular pertussis vaccines on the evolving epidemiology of pertussis that has been observed over the past decade.

## Background and Epidemiology of Tetanus

Tetanus is a life-threatening but vaccine-preventable disease caused by a potent neurotoxin produced by *Clostridium tetani*. The organism is a ubiquitous, spore-forming, motile Gram-positive bacillus found in high concentrations in soil and animal excrement. *C. tetani* spores enter the body through breaches in the skin or mucous membranes. Germination of *C. tetani* spores occurs under anaerobic conditions, such as in necrotic tissue that can result from deep puncture wounds or blunt trauma. *C. tetani* bacilli vegetate and produce tetanospasmin, a powerful exotoxin that binds irreversibly with neural tissue and causes spasms and rigidity of skeletal muscles. Direct person-to-person transmission of *C. tetani* does not occur (*45*).

The incubation period from injury to symptom onset varies from 3 to 21 days (median: 7 days), with extremes of 1 day to several months. The incubation period depends on the severity and site of the wound. Shorter incubation periods are associated with more severe disease and a poorer prognosis; longer incubation periods are associated with injuries furthest from the central nervous system. The course of disease is variable but is usually intense for ≥4 weeks before subsiding. The convalescent period is usually protracted and long-term neurologic sequelae and intellectual and behavioral abnormalities might follow recovery. The case-fatality ratio for tetanus is highest in infants and the elderly, and can be as high as 100% without high-quality medical care, but is approximately 10%–20% even in modern health care facilities (*46*).

### Epidemiology of Tetanus in the United States

**FIGURE 3:**
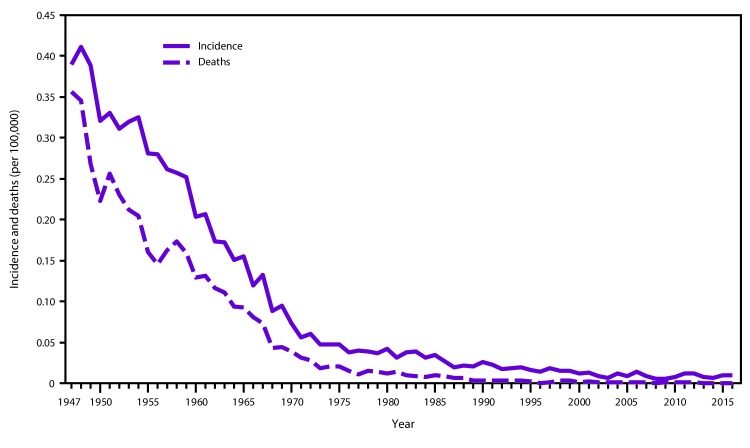
Annual incidence* of and deaths attributable to tetanus — United States, 1947–2016 **Sources:** National Notifiable Diseases Surveillance System and passive reports to the U.S. Public Health Service. * Per 100,000 population.

Tetanus is a nationally notifiable disease in the United States (*15*). After the introduction of universal vaccination with tetanus toxoid–containing (TT) vaccines in the mid-1940s, the incidence of reported tetanus in the United States declined by >98%, from 0.39 per 100,000 population in 1947, when national reporting began, to 0.01 per 100,000 population by 2016 (CDC, unpublished data, 2016) (Figure 3). The decline in incidence occurred across all age groups. Deaths from tetanus also declined similarly during this period. The decline in morbidity and mortality is attributable to widespread use of tetanus toxoid–containing vaccines; improved wound management, including use of tetanus prophylaxis in emergency departments; improved hygiene during childbirth; increased levels of maternal immunity; and expanded urbanization (*45*).

Tetanus occurs primarily among older adults (*47*). During 2001–2016, three neonatal tetanus cases and 459 non-neonatal tetanus cases were reported to the National Notifiable Diseases Surveillance System (NNDSS). The median age for non-neonatal cases was 44.0 years (range: 2–95 years); 60% of cases occurred in males (CDC, unpublished data, 2016). The risk for both tetanus disease and mortality was higher among persons aged ≥65 years than among persons aged <65 years (*48*). Tetanus occurs almost exclusively among persons who are unvaccinated or inadequately vaccinated or in those whose vaccination histories are unknown or uncertain. The case-fatality ratio for reported tetanus in the United States declined from 18% (1998–2000) to 8.0% (2001–2016) (CDC, unpublished data, 2016) (*48*,*49*).

### Population Immunity

The minimum level of circulating antitetanus antibodies associated with protection against tetanus is assay-specific. The acceptable level of circulating antitetanus antibodies required for protection is 0.01 IU/mL as measured in an in vivo toxin neutralization assay. When in vitro methods, such as standard enzyme-linked immunosorbent assays (ELISA), are used, antibody level readings of at least 0.1–0.2 IU/mL are considered protective (*50*).

The National Health and Nutritional Examination Survey (NHANES III), a population-based national serosurvey conducted in the United States during 1988–1994, found that approximately 80% of adolescents aged 12–19 years and >80% of adults aged 20–39 years had seroprotective concentrations of antitetanus toxoid antibodies (*51*). In this survey, a standard ELISA test was used to assess antibody levels with levels >0.15 IU/mL considered as protective. The proportions of persons lacking protective levels of circulating antibodies against tetanus toxin increased with age, with a greater rate of decline among women. By age 70 years, only 45% of men and 21% of women had a protective level of antibodies to tetanus. Previous military service was associated with a higher prevalence of protective antibodies to tetanus in men, presumably because of routine vaccination during military service. The low prevalence of detectable antibodies and the high proportion of tetanus cases among older adults reflects the high proportion of older adults who possibly never received primary DTP vaccination or have waning immunity if they never received subsequent tetanus toxoid–containing booster doses (*48*,*51*).

### Prevention

Immunity to tetanus toxin is rarely if ever acquired naturally, but tetanus is preventable through the use of highly effective tetanus toxoid–containing vaccines (i.e., DTaP, DT, Td, or Tdap) (*50*). Completing a 5-dose childhood vaccination series with DTaP before age 7 years is necessary for developing protective levels of antitetanus antibodies that persist into the adolescent years, when a booster dose of vaccine is needed; thereafter, decennial boosters with Td administered throughout adulthood are recommended to maintain protection against tetanus (*52*).

## Background and Epidemiology of Diphtheria

Respiratory diphtheria is an acute, communicable infectious illness caused by toxigenic strains of *Corynebacterium diphtheriae*, which are nonmotile, nonencapsulated, club-shaped, Gram-positive bacilli. Although rare, toxin-producing *Corynebacterium ulcerans* can also cause a diphtheria-like illness (*53*). Vaccination with diphtheria toxoid-containing vaccines (i.e., DTaP, DT, Tdap, or Td) prevents diphtheria (*54*). Toxin-producing strains of *C. diphtheriae* can cause disease in susceptible persons by multiplying and producing diphtheria toxin in either nasopharyngeal or skin lesions. The classic feature of respiratory diphtheria is a gray-colored pseudomembrane that is firmly adherent to the mucosa lining the nasopharynx, tonsils, or larynx. The extension of the pseudomembrane into the trachea-bronchial tree might cause life-threatening airway obstruction. In addition, systemic absorption and dissemination of diphtheria toxin can cause toxin-mediated cardiac and neurologic complications (*55*).

### Epidemiology of Diphtheria in the United States

**FIGURE 4:**
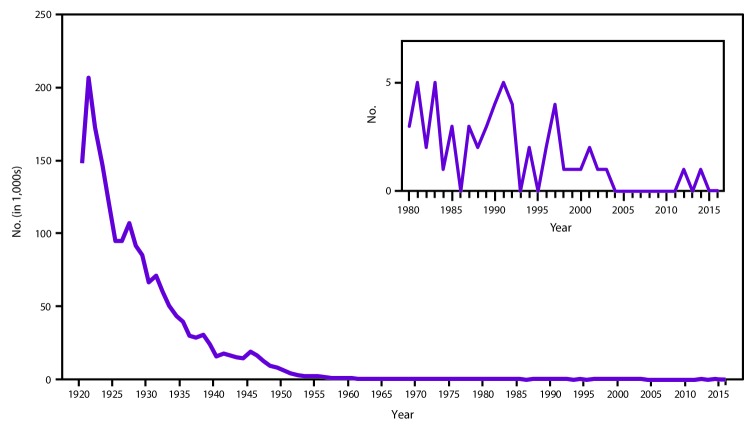
Number of reported diphtheria cases — United States, 1920–2016 **Sources:** National Notifiable Diseases Surveillance System and passive reports to the U.S. Public Health Service.

In the United States, diphtheria is a nationally notifiable disease (*15*). Reported diphtheria cases from all anatomical sites declined from approximately 200,000 in 1921 to 15,536 in 1940 (Figure 4). This decline continued after the introduction of universal childhood vaccination in the late 1940s and, in 1980, only two cases of diphtheria were reported (*56*). Since 1980, cutaneous diphtheria has not been reportable and only respiratory cases are reportable to NNDSS. During 1996–2016, a total of 13 cases were reported (CDC, unpublished data, 2016) (*21*–*23*,*57*–*63*). Although no cases were reported during 2004–2011, a probable case with a positive polymerase chain reaction (PCR) test for the diphtheria toxin gene (“tox”) occurred in 2012, and a case positive by culture with nontoxigenic *C. diphtheriae* was reported in 2014 (*24*,*47*).

Although childhood DTaP vaccination coverage is >80% in the United States, immunity acquired from childhood vaccination wanes in the absence of decennial boosters, and older adults might not be adequately protected (*51*). An analysis of NHANES III indicated that only 60% of the overall population sample had immunity to diphtheria (defined as an antidiphtheria toxoid concentration of >0.1 IU/mL). This immunity declined progressively with increasing age from 91% at age 6–11 years and 80% among adolescents aged 12–19 years, to approximately 30% among those aged 60–69 years (*54*). Data from the 2015 National Immunization Survey (NIS) indicated that 61.6% of adults aged 19–49 years received any tetanus toxoid–containing vaccination during the preceding 10 years, 64.1% (aged 50–64 years), and 56.9% (aged ≥65 years) (*36*).

Although rare in the United States, exposure to diphtheria remains possible during travel to countries with endemic disease^†^ or from imported cases (*64*). Information about the clinical management of diphtheria, including use of diphtheria antitoxin, and the public health response is available at https://www.cdc.gov/diphtheria/clinicians.html.

## Vaccines for Prevention of Pertussis, Tetanus, and Diphtheria

Vaccines of different compositions, formulations, and combinations are licensed and available in the United States for different age groups to prevent pertussis, tetanus, and diphtheria (Tables 4 and 5). The indication and age for vaccination might differ by vaccine product and licensure. In certain situations, the off-label use of Tdap vaccine has been recommended, including the absence of a minimum interval between the last tetanus toxoid–containing vaccine and receipt of Tdap, catch-up vaccination for those aged 7–10 years, and vaccination of women during each pregnancy (*6*,*8*).

**TABLE 4:** Composition of vaccines containing tetanus toxoid, diphtheria toxoid, and acellular pertussis antigens and age for approved use by vaccine type for persons aged less than 7 years — United States, 2017

Vaccine type	Trade name	Manufacturer	Pertussis antigens (*μ*g)				Diphtheria toxoid (Lf)	Tetanus toxoid (Lf)	Age for approved use in routine and catch-up immunization schedules				
			PT	FHA	PRN	FIM			2 mos	4 mos	6 mos	15–18 mos	4–6 yrs
DTaP vaccines*													
DTaP	Infanrix	GlaxoSmithKline	25	25	8		25	10	X^†^	X	X	X	X
DTaP	Daptacel	Sanofi Pasteur, Inc.	10	5	3	5	15	5	X^†^	X	X	X	X
Combination vaccines with DTaP*													
DTaP-IPV-HepB	Pediarix	GlaxoSmithKline	25	25	8		25	10	X^†^	X	X	X	X^§^
DTaP-IPV-Hib	Pentacel	Sanofi Pasteur, Inc.	20	20	3	5	15	5	X^†^	X	X	X	X^¶^
DTaP-IPV	Kinrix	GlaxoSmithKline	25	25	8		25	10					X
DTaP-IPV	Quadracel	Sanofi Pasteur, Inc.	20	20	3	5	15	5					X
DT vaccine*													
DT	No trade name	Sanofi Pasteur, Inc.					6.7	5	X^†^	X	X	X	X

**Abbreviations**: DT= diphtheria and tetanus toxoids vaccine; DTaP = diphtheria and tetanus toxoids and acellular pertussis vaccine; FDA= U.S. Food and Drug Administration; FHA = filamentous hemagglutinin; FIM = fimbriae types 2 and 3; HepB = hepatitis B; Hib = *Haemophilus influenza* type b; IPV = inactivated poliovirus; Lf = limit of flocculation unit; PRN = pertactin; PT = pertussis toxin.

* Vaccine dosage and administration: 0.5mL intramuscular injection.

^†^ FDA-approved for use in infants as young as 6 weeks.

^§^ FDA-approved for use through age 6 years (prior to 7th birthday).

^¶^ FDA-approved for use through age 4 years (prior to 5th birthday).

**TABLE 5:** Composition of vaccines containing tetanus toxoid, diphtheria toxoid, and acellular pertussis antigens and age for approved use by vaccine type for persons aged ≥7 years — United States, 2017

Vaccine type	Trade name	Manufacturer	Age (yrs) for approved use in routine and catch-up immunization schedules	Pertussis antigens (*μ*g)				Diphtheria toxoid (Lf)	Tetanus toxoid (Lf)
				PT	FHA	PRN	FIM		
Tdap vaccines*									
Tdap	Adacel	Sanofi Pasteur, Inc.	10–64	2.5	5	3	5	2	5
Tdap	Boostrix	GlaxoSmithKline	≥10	8	8	2.5		2.5	5
Td vaccines*									
Td	No trade name	MassBiologics	≥7					2	2
Td	Tenivac	Sanofi Pasteur, Inc.	≥7					2	5

**Abbreviations**: FHA = filamentous hemagglutinin; FIM = fimbriae types 2 and 3; Lf = limit of flocculation unit; PRN = pertactin; PT = pertussis toxin;Td = tetanus and diphtheria toxoids vaccine; Tdap = tetanus toxoid, reduced diphtheria toxoid vaccine, and acellular pertussis vaccine.

* Vaccine dosage and administration: 0.5mL intramuscular injection.

### Tetanus Component

TT vaccine became commercially available in the United States in 1938. After the 1940s, tetanus toxoid was available in combination with diphtheria toxoid with or without whole-cell pertussis antigens in vaccines. Although single antigen TT was used predominantly before 1960, use of Td has replaced TT; production of TT was discontinued in 2013.

In the United States, manufactured tetanus toxoid is adsorbed to aluminum salt adjuvants (aluminum hydroxide, aluminum phosphate, or aluminum sulfate), thimerosal-free, and highly purified, with <0.02% formaldehyde and <1.25 mg aluminum. Pediatric formulations of tetanus toxoid–containing vaccines (DT and DTaP) contain 5–10 limit of flocculation (Lf) units of the antigen (Table 4). Adolescent and adult formulations (Td and Tdap) contain ≤5 Lf units of tetanus toxoid per 0.5 ml dose (Table 5). More than a single dose of vaccine is required to induce immunologic protection, and booster doses are required to maintain protection.

#### Immunogenicity and Effectiveness

Although no randomized controlled clinical trial of the efficacy of tetanus toxoid in preventing disease ever has been conducted, evidence from observational studies consistently supports the effectiveness of vaccination. The incidence of tetanus among U.S. army personnel declined from 13.4 per 100,000 during World War I (when personnel were unvaccinated) to 0.44 per 100,000 during World War II (when personnel routinely were vaccinated with TT and also vaccinated following an injury) (*65*). Similar observations were made among British army personnel during the same periods during the two world wars (*66*). The effectiveness of tetanus toxoid is very high, although not 100%. Although data on a minimum antitetanus antibody cut-off level for protection are sparse, it is generally accepted that levels at 0.01 IU/mL and above by in vivo toxin neutralization assay are protective (*46*,*54*). One dose of tetanus toxoid vaccine provides little, if any, immunity. After receiving 3 doses of tetanus toxoid–containing vaccine, virtually all infants and adults develop protective tetanus antitoxin titers >0.1 IU/mL. A primary immunization series with 3 doses of tetanus toxoid induced a mean antitetanus level of 0.2 IU/mL, and antibody levels from primary vaccination provide protection from tetanus for approximately 3–5 years (*67*–*69*). Additional booster tetanus doses heighten the immune response and prolong the duration of protective immunity. Booster doses at age 4–8 years and during adolescence provide long-lasting protective immunity and a duration of 20–30 years from the last dose has been suggested (*50*).

### Diphtheria Component

Diphtheria toxoid was shown to be immunogenic in 1923 and has since been used as the immunizing agent against diphtheria (*70*). The immunogenicity of diphtheria toxoid is improved when it is adsorbed onto an adjuvant (most commonly aluminum hydroxide or aluminum phosphate). By the mid-1940s, diphtheria toxoid was combined with tetanus toxoid and pertussis vaccine as DTP and later adsorbed onto an aluminum salt and used in the routine childhood vaccination program. Only vaccines containing formaldehyde-inactivated diphtheria toxin adsorbed to an aluminum salt adjuvant combined with tetanus toxoid with or without acellular pertussis vaccines are available in the United States (Tables 4 and 5). More than a single dose of vaccine is required to induce immunologic protection, and booster doses are required to maintain protection (*71*).

#### Immunogenicity and Effectiveness

Although no randomized controlled clinical trial of the efficacy of diphtheria toxoid in preventing disease has ever been conducted, strong evidence from observational studies supports the effectiveness of vaccination (*72*). The effectiveness of diphtheria toxoid is high, although not 100%. After receiving 3 doses of diphtheria toxoid–containing vaccines, virtually all infants develop diphtheria antitoxin titers >0.01 IU/mL (*71*,*73*,*74*). Although some DTaP products produce lower geometric mean titers than those observed after vaccination with DTP, these differences are not thought likely to be clinically significant. For primary vaccination of adults aged ≥19 years, data suggest that virtually all adults develop diphtheria antitoxin titers >0.01 IU/mL after receiving 3 doses of diphtheria toxoid–containing vaccines (*75*,*76*). A diphtheria antitoxin level of 0.01 to 0.09 IU/mL provides some degree of protection, whereas levels ≥0.1 IU/mL are considered protective and levels >1.0 IU/mL are associated with long-lasting protection (*77*). Although no level of circulating diphtheria antitoxin confers absolute protection, most reports indicate that *C. diphtheriae* infection in previously vaccinated persons is milder and less likely to be fatal (*56*,*78*,*79*). The failure of the vaccine to protect all persons exposed to *C. diphtheriae* highlights the importance of maintaining high vaccination coverage and herd immunity to prevent or limit transmission and outbreaks, as evidenced by the disappearance of diphtheria cases in industrialized countries with established vaccination programs.

Although various schedules used worldwide for primary vaccination (3 doses during infancy or 4 doses by age 15 months) appear to provide adequate protection from diphtheria in the early years of life, a booster dose is needed at age 4–6 years to maintain protection throughout the school-age years (*71*). The massive epidemic in the former Union of Soviet Socialist Republics in the 1990s strongly suggests that sustaining high vaccination coverage with a primary series of diphtheria toxoid–containing vaccine among infants and administering booster doses at school entry and throughout life are important for maintaining population immunity (*80*). In developed countries where diphtheria is well controlled, there is little to no opportunity for exposure and natural boosting of immunity from infection after childhood. The World Health Organization (WHO) recommends that persons living in areas of low endemicity or areas where disease is not endemic should receive booster doses of combined diphtheria and tetanus toxoids approximately 10 years after completing the primary series and subsequently every 10 years throughout life (*70*).

### Tetanus and Diphtheria Toxoid–containing Vaccines

As of 2016, one DT vaccine product and two Td vaccine products are licensed by FDA and available in the United States (Tables 4 and 5); production of TT vaccine was discontinued in 2013, and it is no longer available in the United States.

#### DT

DT product (no trade name; Sanofi Pasteur, Swiftwater, Pennsylvania) is licensed by FDA for active vaccination against diphtheria and tetanus in children up to age 7 years for whom the pertussis vaccine component is contraindicated or in situations when the health care provider decides that pertussis vaccine should not be administered. The concentration of diphtheria toxoid is higher in DT vaccine compared to Td vaccine. Additional information is available in the package insert (https://www.fda.gov/downloads/BiologicsBloodVaccines/Vaccines/ApprovedProducts/UCM142732.pdf).

#### Td

Tenivac (Sanofi Pasteur, Swiftwater, Pennsylvania) is licensed by FDA as a booster vaccination against tetanus and diphtheria in persons aged ≥7 years. Additional information is available in the package insert (https://www.fda.gov/downloads/BiologicsBloodVaccines/Vaccines/ApprovedProducts/UCM152826.pdf).

Td product (no trade name; manufactured by MassBiologics, Boston, Massachusetts; distributed by Grifols, Los Angeles, California) is licensed by FDA for active vaccination for the prevention of tetanus and diphtheria in persons aged ≥7 years. Additional information is available in the package insert (https://www.fda.gov/downloads/BiologicsBloodVaccines/Vaccines/ApprovedProducts/UCM164127.pdf).

### Acellular Pertussis Components

In the United States, all vaccines available for preventing pertussis are acellular pertussis formulations combined with tetanus and diphtheria toxoids. Depending on the manufacturer, the pertussis antigens included in acellular pertussis vaccines are: pertussis toxin (PT), filamentous haemagglutinin (FHA), pertactin (PRN) and/or fimbriae types 2 and 3 (FIM). The amount of pertussis antigens present differs depending on the vaccine type and manufacturer (Tables 4 and 5). No “pertussis-only” vaccines are licensed in the United States.

#### Interpretation of Immunogenicity Data

Vaccine efficacy studies have demonstrated a correlation between the presence of antipertussis antibodies and protection against pertussis disease, but there are no well-accepted definitive serologic or laboratory correlates of protection against pertussis (*81*–*85*). Antibody studies are useful for comparing the immune responses elicited by a single vaccine under different conditions or in different studies, whereas efficacy studies are important to measure clinical protection conferred by each pertussis vaccine (*54*).

Licensure of new pertussis vaccines is based on the overall safety profile and the demonstration of immunogenicity not inferior to U.S.-licensed pediatric DTaP products in clinical trials (*86*). In a noninferiority trial, immunogenicity, efficacy, or safety endpoints are demonstrated when a new product is at least as good as a comparator on the basis of a predefined and narrow margin for a clinically acceptable difference between the study groups (*87*).

### DTaP Vaccines

DTaP vaccines consist of pertussis antigens and diphtheria and tetanus toxoids (Table 4). Depending on vaccine type and manufacturer, the composition and amount of pertussis antigen and amount of diphtheria and tetanus toxoids differs. The FDA-approved age indication for use of DTaP vaccines differs, depending upon the specific DTaP product (Table 4). Data on immunogenicity and safety of DTaP vaccines have been published (*3*,*88*–*94*).

#### Licensed and Available DTaP Vaccines

As of 2016, two DTaP vaccines are licensed by FDA and available in the United States: Infanrix (GlaxoSmithKline [GSK], Rixensart, Belgium) and Daptacel (Sanofi Pasteur, Swiftwater, Pennsylvania). Immunogenicity and safety data for each of these vaccines have been published (*3*,*88*,*89*,*91*).

Infanrix (GSK) is licensed by FDA for active vaccination against diphtheria, tetanus, and pertussis as a 5-dose series in infants and children aged 6 weeks through 6 years. Additional information is available in the package insert (https://www.fda.gov/downloads/BiologicsBloodVaccines/Vaccines/ApprovedProducts/UCM124514.pdf).

Daptacel (Sanofi Pasteur) is licensed by FDA for active vaccination against diphtheria, tetanus, and pertussis as a 5-dose series in infants and children aged 6 weeks through 6 years. Additional information is available in the package insert (https://www.fda.gov/downloads/BiologicsBloodVaccines/Vaccines/ApprovedProducts/UCM103037.pdf).

#### Licensed and Available Combination Vaccines That Include DTaP

As of 2016, four combination vaccines that contain the components of DTaP vaccines are licensed by FDA and available in the United States: DTaP-IPV-HepB (Pediarix, GSK, Rixensart, Belgium), DTaP-IPV (Kinrix, GSK, Rixensart, Belgium), DTaP-IPV/Hib (Pentacel, Sanofi Pasteur, Swiftwater, Pennsylvania), and DTaP-IPV (Quadracel, Sanofi Pasteur, Swiftwater, Pennsylvania). Combination vaccines with DTaP have been shown to be both safe and immunogenic, and have similar safety profiles and antibody responses compared with DTaP administered by itself (*95*–*97*).

DTaP-IPV-HepB (Pediarix) contains DTaP, inactivated poliovirus (IPV), and Hepatitis B (recombinant) (HepB). Pediarix is approved by FDA for use as a 3-dose series in infants born to hepatitis B surface antigen (HBsAg)-negative mothers. Pediarix can be administered as early as age 6 weeks through 6 years. Additional information is available in the package insert (https://www.fda.gov/downloads/BiologicsBloodVaccines/Vaccines/ApprovedProducts/UCM241874.pdf).

DTaP-IPV (Kinrix) contains DTaP and IPV. Kinrix is licensed by FDA for use as the fifth dose of the DTaP vaccine series and the fourth dose of the IPV series in children aged 4–6 years whose previous DTaP vaccine doses were DTaP (Infanrix, GSK) and/or DTaP-HepB-IPV (Pediarix, GSK) for the first 3 doses and DTaP (Infanrix) for the fourth dose. Additional information is available in the package insert (https://www.fda.gov/downloads/BiologicsBloodVaccines/Vaccines/ApprovedProducts/UCM241453.pdf).

DTaP-IPV/Hib (Pentacel) contains DTaP, IPV, and *Haemophilus influenzae* type b (Hib) conjugate. Pentacel is licensed by FDA for use as a 4-dose series in children aged 6 weeks through 4 years. Additional information is available in the package insert (https://www.fda.gov/downloads/BiologicsBloodVaccines/Vaccines/ApprovedProducts/UCM109810.pdf).

DTaP-IPV (Quadracel) contains DTaP and IPV. Quadracel is licensed by FDA for use as the fifth dose of the DTaP vaccine series and the fourth or fifth dose of the IPV series in children aged 4 through 6 years who have previously received 4 doses of Pentacel and/or Daptacel vaccine. Further information is available in the package insert (https://www.fda.gov/downloads/BiologicsBloodVaccines/Vaccines/ApprovedProducts/UCM439903.pdf).

### DTaP Vaccine Immunogenicity, Efficacy, and Effectiveness

#### Immunogenicity of DTaP Vaccines

**Infanrix (GSK):** One month after receiving 3 doses of Infanrix at ages 2, 4, and 6 months, ≥83% of children had a fourfold or greater antibody response to PT, FHA, and PRN (*98*). All children developed diphtheria antitoxin titers of ≥0.1 IU/mL and tetanus antitoxin titers of ≥0.01 IU/mL (i.e., indications of immunity against these diseases) (*3*). Whether the first 3 doses were Infanrix or DTP, >80% of children aged 15–20 months had a fourfold or greater rise in serum antibody to each of the pertussis vaccine antigens after a fourth dose of Infanrix (*99*). Immunogenicity data on the fifth dose were not required for FDA approval (*100*).

**Daptacel (Sanofi Pasteur):** After 4 doses of Daptacel, the antibody response to pertussis antigens among U.S. infants was similar to that achieved among Swedish infants in whom efficacy was demonstrated after receiving 3 doses of Daptacel (*88*,*101*). Diphtheria antitoxin levels of ≥1.0 IU/mL were achieved by 98.5% of children, and 100% of children achieved tetanus antitoxin levels of ≥1.0 IU/mL (*101*). Licensure for use of Daptacel as a fifth dose at age 4–6 years was based on the assumption that children previously primed with this vaccine will have a robust immune response to a booster dose of the same vaccine. For diphtheria and tetanus, it was expected that most children will have protective levels of antibody following booster vaccination (*102*).

#### Immunogenicity of Combination Vaccines with DTaP

**Pediarix (GSK) (DTaP-HepB-IPV):** The immunologic response of all antigens in Pediarix (diphtheria and tetanus toxoids; pertussis antigens; Hepatitis B virus; and inactivated poliovirus Types 1, 2, and 3) following 3 doses at age 2, 4, and 6 months was generally similar to those following 3 doses of separately administered Infanrix [DTaP (GSK)], ENGERIX-B (HepB), and oral poliovirus vaccine (*89*).

**Kinrix (GSK) (DTaP-IPV):** The immunogenicity of all antigens in Kinrix (diphtheria and tetanus toxoids; pertussis antigens; and inactivated poliovirus Types 1, 2, and 3) was similar between groups (DTaP-IPV and separately administered DTaP and IPV vaccines) with or without a co-administered second dose of measles, mumps, and rubella (MMR) vaccine (*92*).

**Pentacel (Sanofi Pasteur) (DTaP-IPV/Hib):** The immunologic response of all antigens in Pentacel (diphtheria and tetanus toxoids; pertussis antigens; inactivated poliovirus types 1, 2, and 3; and *Haemophilus influenzae* Type b conjugate) following 3 or 4 doses generally was similar to those following separately administered component vaccines (*86*,*103*). Immune responses following the first and second doses were not measured (*93*).

**Quadracel (Sanofi Pasteur) (DTaP-IPV):** The immunogenicity of all antigens in Quadracel (diphtheria and tetanus toxoids; pertussis antigens; and inactivated poliovirus types 1, 2, and 3) was noninferior between groups (DTaP-IPV and separately administered DTaP [Daptacel] and IPV [IPOL, Sanofi Pasteur] vaccines) with or without a co-administered second dose of MMR and varicella vaccines (*94*).

#### Pertussis Vaccine Efficacy

The efficacy of both DTaP vaccine products (Infanrix [GSK] and Daptacel [Sanofi Pasteur]) was evaluated in prelicensure trials in which participants received a 3-dose series at ages 2, 4, and 6 months (*104*–*106*). The vaccine efficacy estimates for 3 doses of DTaP against pertussis disease^§^ ranged from 79% to 89%, with a follow-up time up to 2 years after receipt of the third dose (*104*–*106*).

#### Postlicensure Pertussis Vaccine Effectiveness

Assessment of the 5-dose DTaP series indicated that the estimated overall effectiveness of the pertussis vaccine was 88.7% (95% confidence interval [CI] = 79.4%–93.8%); within the first year after the fifth DTaP dose, vaccine effectiveness was 98.1% (95% CI = 96.1%–99.1%) (*28*). However, vaccine effectiveness declined with increasing time since receipt of the fifth DTaP dose; by ≥5 years since the fifth DTaP dose, vaccine effectiveness was estimated at 71.2% (95% CI = 45.8%–84.8%) (*28*). Other studies support the findings of a progressive decrease in DTaP vaccine effectiveness and increased risk for pertussis over time after receipt of the fifth dose (*107*,*108*). In contrast, an early assessment of DTaP indicated 100% vaccine effectiveness against pertussis for a limited period of time after receipt of 5 doses in children up to age 5 years, but this assessment was done shortly after ACIP issued the 5-dose DTaP recommendation and the majority of participants had received DTP as the first 3 doses (*109*).

### Postlicensure Safety Surveillance of DTaP

Studies conducted since the introduction of acellular pertussis vaccines in the United States have supported the safety of DTaP (*110*–*123*). A summary of these studies is available at https://stacks.cdc.gov/view/cdc/52822. Many of these studies were performed through surveillance for adverse events following vaccine receipt through two systems in the United States, Vaccine Adverse Event Reporting System (VAERS) and Vaccine Safety Datalink (VSD). VAERS is a national passive surveillance system operated jointly by CDC and FDA that receives reports of adverse events following vaccination from health care personnel, manufacturers, vaccine recipients, and others (*124*). VSD is a collaborative effort of CDC and eight managed care organizations in the United States and allows for planned vaccination safety studies and timely investigation of hypotheses that arise from the review of the medical literature, reports to VAERS, changes in the vaccination schedule, or the introduction of new vaccines (*125*).

#### Safety of Licensed and Available DTaP Vaccines in VAERS

During January 1, 1990–July 31, 2015, VAERS received 46,448 reports involving receipt of one of the five DTaP vaccines that are available in the United States during that period (Daptacel, Infanrix, Kinrix, Pediarix, and Pentacel); 44,061 (95%) of the reports involved children aged <6 years. DTaP vaccine was administered concurrently with one or more other vaccines in 40,868 (88%) case reports (CDC, unpublished data, 2016). The median time from vaccination to onset of an adverse event was 1 day. The most frequently reported adverse events were injection-site erythema (11,879 [26%]), pyrexia (9,225 [20%]), injection-site swelling (6,964 [15%]), erythema site other than injection site or site not specified (5,339 [12%]), and injection-site warmth (4,468 [10%]). When VAERS DTaP reports for each vaccine brand were compared individually with those for all other inactivated vaccines in the VAERS database, no concerning patterns of adverse events were observed.

Among all DTaP vaccine-related reports, 5,205 (11%) were coded as serious (i.e., one of the following outcomes was reported: death, life-threatening illness, hospitalization, prolongation of hospitalization, or permanent disability). Among those reports coded as serious, the most frequent adverse events were pyrexia (1,795 [35%]), vomiting (1,420 [27%]), irritability (1,101 [21%]), seizure (938 [18%]), and intussusception (746 [14%]). In 97% of the 728 intussusception reports, rotavirus vaccine was administered concomitantly. Intussusception has been associated with administration of both U.S.-licensed rotavirus vaccine products (*126*,*127*).

A total of 793 deaths following receipt of DTaP vaccines were reported in VAERS during the study period (CDC, unpublished data, 2016). An autopsy report or other type of medical record was available for 682 (86%) reports and reviewed for cause of death. The most frequent reported cause of death was sudden infant death syndrome (SIDS) in 338 (49.6%) reports. Other categories of death included asphyxiation (47 [6.8%]); diseases of the respiratory system (44 [6.5%]); diseases of the circulatory system (27 [3.9%]); certain infections or parasitic diseases (27 [3.9%]); diseases of the nervous system (24 [3.5%]); and congenital malformations, deformations and chromosomal abnormalities (23 [3.4%]). In 90 (13.2%) death reports, the cause was undetermined and in 62 (9.1%) death reports various other causes were reported (e.g., blunt force trauma). These reported frequencies are similar to those observed with overall U.S. infant mortality data and among recipients of other recommended childhood vaccines (*128*). Two recent VSD studies do not suggest a causal relation or increased risk for death following vaccination of any type (*129*,*130*).

#### Adverse Events Associated with Vaccines with Pertussis Components or Tetanus Toxoid–Containing Components

##### Vaccines with Pertussis Components

Because of concerns about the possible role of vaccines with acellular pertussis components in causing neurologic reactions or exacerbating underlying neurologic conditions, ACIP recommendations to defer pertussis vaccines in infants with suspected or evolving neurologic disease, including seizures, have been based primarily on concerns that neurologic events after vaccination (with whole-cell preparations in particular) might interfere with the subsequent evaluation of the infant’s neurologic status (*3*,*12*,*131*).

During the whole-cell pertussis vaccine era, the Institute of Medicine (IOM) concluded that evidence favored acceptance of a causal relation between pediatric DTP use and acute encephalopathy (*132*). After the change to DTaP vaccines, IOM reviewed the evidence for a causal association between acellular pertussis–containing vaccines and several neurologic outcomes (*133*). The evidence was inadequate to accept or reject a causal relation between receipt of acellular pertussis-containing vaccine and encephalitis, encephalopathy, infantile spasms, seizures, ataxia, autism, acute disseminated encephalomyelitis, transverse myelitis, optic neuritis, onset of multiple sclerosis in adults, relapse of multiple sclerosis in adults, relapse of multiple sclerosis in children, Guillain-Barré syndrome, chronic inflammatory disseminated polyneuropathy, opsoclonus myoclonus syndrome, or Bell’s palsy (*133*).

Pediatric DTaP is contraindicated in children with a history of encephalopathy not attributable to another identifiable cause occurring within 7 days after pediatric DTP/DTaP vaccination (Table 2). Although active surveillance in Canada among a population of children administered 6.5 million doses of pertussis vaccines during 1993–2002 failed to ascertain any acute encephalopathy cases causally related to whole-cell or acellular pertussis vaccines, postlicensure surveillance in Japan during a 23 year period demonstrated rates of encephalopathy/encephalitis (death) of 7.6 cases within 7 days of vaccination per 10 million doses during 1970–1974 when DTP was administered, and 0.5 cases per 10 million doses during 1989–2000 when DTaP replaced DTP (*113*,*134*).

ACIP recommends that infants with evolving neurologic conditions not be vaccinated with pediatric DTaP until a treatment regimen has been established and the condition has stabilized (Table 2) (*3*). A history of seizures (febrile or afebrile) <3 days after a previous dose of DTP/DTaP, a history of well-controlled seizures in the vaccinee or a family history of seizures or other neurologic disorder is not a contraindication or precaution to vaccination with pertussis components (Table 3) (*3*).

Hypotonic-hyporesponsive episodes (HHE) and prolonged crying are adverse events that are less commonly reported with DTaP than were historically reported with DTP (*111*,*135*,*136*). Neither HHE nor prolonged crying after receipt of DTP/DTaP are known to be associated with serious sequelae, and both adverse events have been reported after receipt of vaccines other than DTP/DTaP (*135*,*136*). Among children who received subsequent DTP/DTaP doses, recurrent HHE occurrences are very rarely reported (*135*,*136*). A single, uncomplicated occurrence of either HHE or prolonged crying does not preclude vaccination, and the benefits of vaccination outweigh the risks for additional episodes.

ACIP recommends that vaccine providers and parents evaluate the risks for and benefits of administering subsequent doses of vaccines with pertussis components to young children who after receiving pediatric DTP/DTaP experienced any of the events listed in the table for contraindications and precautions for DTaP, DT, Td, or Tdap vaccines (Table 2). All of these events were documented more frequently following whole-cell pertussis vaccines than following acellular vaccines (*3*,*110*,*111*,*136*,*137*).

##### Tetanus Toxoid–Containing Vaccines

As with the recent conclusions regarding acellular pertussis–containing vaccines, IOM also concluded that the evidence was inadequate to accept or reject a causal relation between receipt of diphtheria toxoid and tetanus toxoid–containing vaccine and encephalitis, encephalopathy, infantile spasms, seizures, ataxia, autism, acute disseminated encephalomyelitis, transverse myelitis, optic neuritis, onset of multiple sclerosis in adults, relapse of multiple sclerosis in adults, relapse of multiple sclerosis in children, Guillain-Barré syndrome, chronic inflammatory disseminated polyneuropathy, opsoclonus myoclonus syndrome, or Bell’s palsy (*133*). ACIP recommends that Guillain-Barré syndrome occurring <6 weeks after receipt of a tetanus toxoid–containing vaccine is a precaution for subsequent administration of tetanus toxoid–containing vaccines (*52*).

IOM has concluded that evidence from case reports and uncontrolled studies involving tetanus toxoid–containing vaccines favored a causal relation between tetanus toxoid–containing vaccines and brachial neuritis (*133*). Although brachial neuritis is considered to be a compensable event through the Vaccine Injury Compensation Program (VICP), ACIP considers that occurrence of brachial neuritis following vaccination with a tetanus toxoid–containing vaccine does not preclude their future use in the same person; brachial neuritis is usually self-limited (*52*,*132*,*138*).

#### Milk Allergy

DTaP and Tdap vaccines might include residual milk allergens from ingredients used during manufacturing (*139*). Because of reports of children and adolescents with a documented history of severe milk allergy having an anaphylactic reaction to booster doses of DTaP or Tdap within one hour of administration (*139*,*140*), a prospective review of VAERS data was conducted. No safety signal in VAERS for anaphylaxis in patients with milk protein allergy was identified, leading to the conclusion that these vaccines are tolerated by those with a milk allergy, and that milk allergy is not a contraindication or precaution to receipt of DTaP or Tdap (*140*); vaccine providers should continue to vaccinate persons with milk allergy as recommended and strongly consider monitoring the patient for anaphylaxis.

### Simultaneous Administration of DTaP with Other Vaccines

Simultaneous administration of vaccines is defined as administering more than one vaccine on the same clinic day, at different anatomical sites, and not combined in the same syringe (*52*). Before the availability of DTaP-containing combination vaccines, administration of DTaP vaccine was recommended with other vaccines on the same clinic day. Limited historic data regarding simultaneous administration of the first 3 doses of DTaP with other childhood vaccines indicate no interference with response to any of these antigens (*3*).

A recent safety study on simultaneous administration of DTaP with other vaccines indicated a small increased risk for febrile seizures during the 24 hours after a child receives the inactivated influenza vaccine (IIV) at the same time as the pneumococcal 13-valent conjugate (PCV13) vaccine or DTaP (*123*). Other studies have not shown an increased risk for febrile seizures after DTaP, except when simultaneously administered with IIV (*114*,*115*,*121*,*122*). The risk for febrile seizure with any combination of these vaccines is small; ACIP recommends simultaneous administration of these vaccines.

### Tdap Vaccines

Two Tdap products are licensed for use in adolescents and adults as a single-dose booster vaccination against tetanus, diphtheria, and pertussis: Boostrix (GlaxoSmithKline, Rixensart, Belgium), and Adacel (Sanofi Pasteur, Swiftwater, Pennsylvania). The age indication for approved use differs depending upon the specific Tdap product and licensure (Table 5). Both Tdap products consist of pertussis antigen and diphtheria and tetanus toxoids (Table 5). The pertussis antigen composition and amount differ, as does the amount of diphtheria toxoids between the two Tdap products. Summaries of the data on the immunogenicity and safety of each of these vaccines have been published (*4*,*5*).

Adacel (Sanofi Pasteur) is licensed by FDA as a single dose in persons aged 10–64 years (*141*). Adacel contains the same tetanus toxoid, diphtheria toxoid, and five pertussis antigens (PT, PRN, FHA, and FIM) as those in Daptacel (pediatric DTaP), but is formulated with reduced quantities of the toxoids and antigens (Table 5). Adacel contains no thimerosal or other preservative. Additional information is available in the package insert (https://www.fda.gov/downloads/BiologicsBloodVaccines/Vaccines/ApprovedProducts/UCM142764.pdf).

Boostrix (GSK) is licensed by FDA as a single dose in persons aged ≥10 years (*142*). Boostrix contains the same tetanus toxoid, diphtheria toxoid, and three pertussis antigens (PT, PRN, and FHA) as those in Infanrix (pediatric DTaP), but is formulated with reduced quantities of the toxoids and antigens (Table 5). Boostrix contains no thimerosal or other preservative. Additional information is available in the package insert (https://www.fda.gov/downloads/BiologicsBloodVaccines/UCM152842.pdf).

#### Immunogenicity and Efficacy

Both Tdap products were licensed on the basis of clinical trials demonstrating immunogenicity not inferior to U.S.-licensed Td or pediatric DTaP products and an overall safety profile clinically comparable to U.S.-licensed Td products (*143*,*144*). Determining the efficacy of the tetanus and diphtheria toxoid components for each Tdap product was based on the comparison of the rates of protective immune response to these antigens as compared to U.S.-licensed Td and using established serologic correlates of protection (*45*,*72*). The percentage of persons achieving protective antitetanus and antidiphtheria antibody concentrations (>0.1 IU/mL) and the booster response to each of these antigens 1 month postvaccination were evaluated.

Because no well-accepted serologic or laboratory correlate of protection against pertussis has been established, clinical endpoint efficacy studies of acellular pertussis vaccines among adolescents or adults were not required for Tdap licensure. Instead, the efficacy of the pertussis components of Tdap vaccines was inferred using a serologic bridge to infants vaccinated with DTaP in efficacy trials with clinical endpoints (*145*). The immune response of adolescents and adults to each pertussis vaccine antigen after a single dose of Tdap was compared with the immune responses of infants who received 3 doses of pediatric DTaP that included the same pertussis components as the Tdap being assessed (*141*,*142*). The percentage of persons with an acceptable booster response to pertussis vaccine antigens according to predefined criteria also was evaluated. The predefined lower limit was defined as the lower limit of 95% CI for the GMC ratio of the Tdap/pediatric DTaP. Prelicensure Tdap vaccine efficacy was inferred using a serologic bridge to infants vaccinated with 3 doses of DTaP and ranged from 79% to 89% (*105*,*106*).

#### Postlicensure Tdap Effectiveness

Following the 2005 Tdap recommendation for adolescents and adults, postlicensure pertussis vaccine effectiveness estimates for Tdap in settings with similar vaccines and recommendation schedules have ranged from 66% to 78% among adolescents who received both DTP and DTaP as children (*146*–*148*). Among adolescents who received only DTaP as children, in a matched case-control study, the overall estimated vaccine effectiveness of Tdap against pertussis was 63.9% (95% CI = 50%–74%) (*29*). Initial vaccine effectiveness against pertussis within one year of Tdap vaccination was 73% (95% CI = 60%–82%), but after 2–4 years, postvaccination vaccine effectiveness decreased to 34% (95% CI = -0.03%–58%) (*29*). Another study that calculated Tdap vaccine effectiveness among adolescents found that, within the first year after vaccination, effectiveness was 68.8% (95% CI = 59.7%–75.9%); by ≥4 years after vaccination, vaccine effectiveness was 8.9% (95% CI = -30.6%–36.4%) (*149*). As observed with DTaP, Tdap vaccine effectiveness declines substantially with increasing time since vaccination (*29*,*149*,*150*). Although there are no studies estimating Tdap vaccine effectiveness in persons who received only DTP as infants, reported rates of pertussis have been observed to be significantly lower among children who had started their vaccination series with DTP than among those who had started with DTaP (*151*,*152*).

#### Prevention of Transmission: Indirect Protection (“Cocooning”)

At the time Tdap was first recommended, it was anticipated that this vaccine would prevent pertussis in adolescents and adults and thereby result in preventing transmission of *B. pertussis* to contacts (e.g., infants). Providing indirect protection through Tdap vaccination to adults was the premise for the “cocooning” strategy to prevent pertussis in young infants at highest risk for severe pertussis morbidity and mortality. A limited number of studies have evaluated the effectiveness of Tdap vaccination in preventing transmission of pertussis in young infants, but the evidence was inconclusive. Although one study found a modest decrease in the risk for pertussis in infants whose mothers received postpartum Tdap, another study found that mother’s postpartum vaccination and cocooning did not reduce pertussis in infants (*153*,*154*).

Studies in animal models have shown that acellular pertussis vaccines protect against disease but not against infection or transmission of *B. pertussis* or the closely related species, *B. bronchiseptica* (*155*–*157*). Although it is unclear if these animal models fully represent human disease, expert opinion is that persons vaccinated with acellular pertussis vaccines can become infected with and transmit *B. pertussis* (*158*,*159*). Persons up to date with pertussis vaccines are less likely to have severe disease compared with those not up to date (*160*). Although it is presumed that vaccinated persons with less severe disease would be less likely to transmit *B. pertussis* because of less frequent or severe coughing, more recent evidence suggests that vaccination with acellular pertussis vaccines does not prevent transmission and therefore does not afford indirect protection against pertussis (*155*–*157*).

#### Postlicensure Safety of Tdap

Since 2005, when both Tdap products were first licensed and recommended, both vaccine product label indications have expanded and ACIP Tdap recommendations have been updated (*6*,*8*–*10*,*161*–*163*). A summary of these recommendations is available at https://stacks.cdc.gov/view/cdc/52821. Routine VAERS surveillance for and VSD studies on adverse events following receipt of Tdap vaccines in persons aged 10–64 years have provided reassuring data that support the prelicensure clinical trial safety data and have not demonstrated any associations between Tdap and the following rare adverse events: encephalopathy-encephalitis-meningitis; paralytic syndromes; seizures; cranial nerve disorders; and Guillain-Barré syndrome (*164*–*166*). Medically attended local reactions were uncommon and did not differ with concomitant or sequential administration of diphtheria toxoid–containing vaccines (Td/Tdap and MenACWY-D [meningococcal serogroups A, C, W, and Y] Menactra, Sanofi Pasteur) (*167*). No increased risk for medically attended neurologic or allergic reactions was observed following Tdap vaccination and, when compared with matched historical Td recipients, no increase in the onset of new chronic illnesses was seen after Tdap (*168*). Although a 2016 study found an increased risk for acute disseminated encephalomyelitis following Tdap vaccination, this finding was based on cases in two Tdap-vaccinated persons and might have been unrelated to vaccination (*169*). Safety data from VAERS, VSD, and other studies in populations not originally routinely recommended to receive Tdap (e.g., adults aged ≥65 years and pregnant women) have become available since 2009 and were reviewed by ACIP (*6*,*8*–*10*).

##### Persons Aged ≥65 Years

For adults aged ≥65 years, Tdap vaccine safety was comparable to that of Td vaccine (*170*,*171*). The most frequent adverse events following receipt of Tdap in persons aged ≥65 years were local injection-site reactions, and no unusual or unexpected clusters of adverse events after Tdap were identified (*170*). In addition, the risks for the following prespecified events were comparable following receipt of Tdap and Td in older persons: meningitis, encephalitis and encephalopathy; cranial nerve disorders, including Bell’s palsy; Guillain-Barré syndrome; brachial neuritis; paralytic syndromes; medically attended inflammatory or allergic events; and anaphylaxis and generalized reactions (*171*).

##### Interval of Tdap After Td

When Tdap was licensed in 2005, the safety of administering a dose of Tdap at intervals <5 years after Td or pediatric DTP/DTaP had not been studied. Evaluations of the safety of administering Tdap at intervals <5 years after Td, including as short as 18 months, suggest that the safety of much shorter intervals is acceptable (*172*–*174*). Two studies were conducted in adults who received a Tdap or combined Tdap-inactivated polio (Tdap-IPV) vaccine <2 years following a previous Td-containing vaccine (*173*,*174*). Observed adverse events were limited to local reactions, including pain (68%–83%), erythema (20%–25%), and swelling (19%–38%) (*173*,*174*). Although serious adverse events did not occur, the numbers of subjects in these studies were small and the potential for rare, but serious, adverse events cannot be excluded. ACIP concluded that although longer intervals between Td and Tdap vaccination could decrease the occurrence of local reactions, the benefits of protection against pertussis outweigh the potential risk for adverse events (*8*).

##### Persons Aged 7–10 Years

ACIP concluded that the overall safety of Tdap and the frequency of local reactions in persons aged 7–10 years who have not completed the childhood DTaP series likely would be similar to those observed in children who received 4 doses of DTaP (*8*). Although both Tdap products are approved for use in persons as young as age 10 years, no data have been published regarding the safety of Tdap in children aged 7–9 years who have never received pertussis-containing vaccines. Several studies assessing the safety and immunogenicity of Tdap or Tdap-IPV as the fifth dose of acellular pertussis vaccine in children aged 4–8 years were reviewed (*175*–*179*). No increase in the risk for severe local reactions or systemic adverse events was observed (*175*–*179*). The most commonly reported adverse events that occurred within 15 days after receipt of Tdap were pain (40%–56%), erythema (34%–53%), and swelling (24%–45%). Fewer local reactions were observed or reported among Tdap or Tdap-IPV recipients compared with those who received DTaP or DTaP-IPV, but the differences were not statistically significant. No differences were noted when children aged 4–6 and 7–8 years were compared with respect to the frequency of solicited or unsolicited adverse reactions following vaccination with Tdap-IPV (*179*).

##### Pregnant Women

In 2011, when ACIP first considered administration of Tdap during pregnancy, safety data on women and their infants were limited (*9*); prelicensure evaluations did not study the safety of administering a booster dose of Tdap to pregnant women. ACIP reviewed available data from VAERS, pregnancy registries established by both Sanofi Pasteur (Adacel) and GSK (Boostrix), and small studies (*174*,*180*–*183*). ACIP concluded that these studies did not suggest any elevated risk for or unusual patterns of adverse events in pregnant women who received Tdap or in their newborn infants, and the few serious adverse events reported were judged unlikely to have been caused by the vaccine (*9*). A summary of these studies is available at https://stacks.cdc.gov/view/cdc/52820.

When ACIP considered recommending Tdap vaccination during each pregnancy, the safety information concerning booster doses of Tdap in pregnant women previously vaccinated with Tdap was not available (*6*). Data on the safety of two closely spaced doses of tetanus toxoid–containing vaccines were limited to receipt of Td and Tdap or Tdap-IPV vaccine within 21 days or ≤2 years and receipt of 2 doses of Tdap at a five-year interval in nonpregnant persons; of the few serious adverse events reported, none were attributed to the vaccine (*172*–*174*,*184*,*185*). Receipt of a second dose of Tdap in nonpregnant persons was well tolerated; injection-site pain was the most commonly reported adverse event (*184*–*188*). The frequency of reported adverse events following the second dose of Tdap was similar to that after the first dose in the same subjects and in controls receiving Tdap for the first time.

A theoretical risk for severe local reactions exists among pregnant women who are vaccinated during multiple closely spaced pregnancies. These severe local reactions are hypersensitivity reactions that have been associated with vaccines containing tetanus toxoid, tetanus, and diphtheria toxoids and/or pertussis antigens in persons who have received multiple doses of vaccine. Most of the data on multiple doses of tetanus toxoid–containing vaccines and hypersensitivity reactions are historical, and the risk for severe adverse events likely has been reduced with current formulations that contain lower concentrations of tetanus toxoid (*45*,*189*,*190*). Recent studies were small and did not include pregnant subjects; therefore, the findings do not exclude the possibility of rare but serious adverse events in pregnant women after receipt of Tdap (*6*).

ACIP recognized the need for safety studies of severe adverse events when Tdap is administered during subsequent pregnancies but concluded that the potential benefit of preventing pertussis morbidity and mortality in infants too young to be fully vaccinated outweighs the theoretical concern of possible localized severe adverse events in pregnant women receiving Tdap. ACIP also concluded that experience with tetanus toxoid–containing vaccines suggests no excess risk for severe adverse events among women receiving Tdap with each pregnancy (*6*).

Additional data from the United States and elsewhere on the safety of Tdap vaccination during pregnancy for both pregnant women and their infants continue to be reassuring, with no reported increase in adverse events, including adverse birth outcomes, and no observation of new or unexpected vaccine safety concerns (*191*–*209*); a summary of these studies is available at https://stacks.cdc.gov/view/cdc/52820. Receipt of Tdap during pregnancy has not been found to be associated with an increased risk for frequency of major malformations, stillbirth, preterm birth, small for gestational age, or hypertensive disorders (*193*–*195*,*197*,*208*). One study observed a slight increase in the risk for chorioamnionitis and, although chorioamnionitis is a risk factor for preterm birth, there were no associated increases in preterm or small for gestational age births in this cohort (*193*). The authors concluded that the small increase in the risk for chorioamnionitis was likely due to residual confounding or heterogeneity in outcome ascertainment (*193*). A review of the VAERS database from 1990 through 2014 found 31 reports of chorioamnionitis following receipt of any vaccine out of 3,389 pregnancy reports (*198*).

An evaluation of the safety of Tdap and influenza vaccines administered concomitantly and sequentially to pregnant women aged 14–49 years found no statistically significant increase in risk for fever or any medically attended acute adverse event in pregnant women vaccinated concomitantly compared with sequentially. No differences in preterm delivery, low birth weight, or small for gestational age neonates were observed between the two groups (*196*).

Data on the safety of receipt of Tdap during pregnancy in close intervals from prior tetanus toxoid–containing vaccinations are limited. One study found no increased risk for acute adverse events (i.e., fever, allergy, and local reactions) or adverse birth outcomes (i.e., small for gestational age, preterm delivery, and low birth weight) for those women who had a previous vaccination ≤5 years before compared with those vaccinated >5 years before receipt of Tdap during pregnancy, suggesting that recent receipt of a prior tetanus toxoid–containing vaccination does not increase risk for adverse events after Tdap vaccination in pregnancy (*195*).

#### Additional Safety Data

##### Neurologic and Systemic Events Associated with Vaccines with Pertussis Components

Concerns about the possible role of vaccines with pertussis components in causing neurologic reactions or exacerbating underlying neurologic conditions are long-standing (*12*,*131*). Although the occurrence of neurologic sequelae after receipt of vaccines with pertussis components is rare, the evidence for a causal association between acellular pertussis–containing vaccines and neurologic outcomes is inconclusive (*133*). Concerns about vaccinating adolescents with progressive or uncontrolled underlying neurologic disease must be weighed against the potential morbidity of pertussis; adolescents with severe neurologic conditions might be at risk for severe pertussis (CDC, unpublished data, 2005) (*39*). ACIP does not consider a history of well-controlled seizures in the vaccinee or a family history of seizures (febrile or afebrile) or other neurologic disorder to be a contraindication or precaution to vaccination with pertussis components (Table 3) (*3*).

ACIP recommends that the risks for and benefits of administering subsequent doses of vaccines with pertussis components be evaluated for young children who, after receiving pediatric DTP/DTaP, experienced any of the events listed in the table as contraindications and precautions for DTaP, DT, Td, and Tdap vaccines (Table 2); all of these events were documented more frequently following whole-cell pertussis vaccines than following acellular vaccines (*3*,*110*,*111*,*136*,*137*). For adolescents and adults, these events (e.g., febrile seizures and HHE) either do not occur or are of less clinical concern than such events in infants and children. Taken together, this information supports administering Tdap to adolescents with a history of the events listed under pediatric DTaP/DTP (Table 2).

ACIP recommends that adolescents with unstabilized progressive neurologic conditions not be vaccinated with Tdap until the condition stabilizes. However, progressive neurologic disorders that are chronic and stable (e.g., dementia) are more common among adults, and the possibility that Tdap would complicate subsequent neurologic evaluation is of less clinical concern. As a result, chronic progressive neurologic conditions that are stable in adults do not constitute a reason to delay Tdap; this is in contrast to unstable or evolving neurologic conditions (e.g., cerebrovascular events and acute encephalopathic conditions) (*5*).

##### Arthus Reactions

Arthus reactions (type III hypersensitivity reactions) are rarely reported after vaccination, but can occur after tetanus toxoid- or diphtheria toxoid–containing vaccines (CDC, unpublished data, 2005) (*54*,*132*,*210*–*214*). An Arthus reaction is a local vasculitis associated with deposition of immune complexes and activation of complement. Immune complexes form in the setting of a high local concentration of vaccine antigens and high concentration of circulating antibody (*210*,*211*,*213*,*215*). Arthus reactions are characterized by severe pain, swelling, induration, edema, hemorrhage, and occasionally necrosis. These symptoms and signs usually occur 4–12 hours after vaccination; by contrast, anaphylaxis (an immediate type I hypersensitivity reaction) usually occurs within minutes of vaccination. As with extensive limb swelling, Arthus reactions usually resolve without sequelae. ACIP recommends that persons who have experienced an Arthus reaction following a dose of tetanus toxoid or diphtheria toxoid–containing vaccine should not receive a tetanus toxoid–containing vaccine more frequently than every 10 years, even for tetanus prophylaxis as part of wound management (*54*).

#### Tetanus Toxoid Safety

Tetanus toxoid is one of the most extensively used vaccines globally, either as a monocomponent vaccine (TT) or combined with diphtheria toxoid (DT and Td) and pertussis antigens (DTP, DTaP, and Tdap). Historically, mild local reactions (i.e., redness, pain and tenderness, and mild swelling) after receipt of TT vaccine are common (0–95%). Systemic reactions (i.e., fever, malaise, headache, and lymphadenopathy) are less common but might occur, particularly after receipt of a booster dose of vaccine. Severe reactions, including neurologic (e.g., peripheral neuropathy, particularly brachial plexus neuropathy, Guillain-Barré syndrome, seizures, and acute encephalopathy) and hypersensitivity reactions (anaphylaxis) are exceedingly rare (*45*,*216*).

An evaluation by IOM concluded that evidence from case reports and uncontrolled studies involving tetanus toxoid–containing vaccines favored a causal relation between tetanus toxoid–containing vaccines and brachial neuritis (*133*). Although brachial neuritis is considered to be a compensable event through the National Vaccine Injury Compensation Program (VICP), ACIP considers that occurrence of brachial neuritis following vaccination with a tetanus toxoid–containing vaccine does not preclude their future use in the same person; brachial neuritis is usually self-limited (*52*,*132*,*138*).

As with the recent conclusions regarding acellular pertussis–containing vaccines, IOM also concluded that the evidence was inadequate to accept or reject a causal relation between receipt of diphtheria toxoid- and tetanus toxoid–containing vaccine and encephalitis, encephalopathy, infantile spasms, seizures, ataxia, autism, acute disseminated encephalomyelitis, transverse myelitis, optic neuritis, onset of multiple sclerosis in adults, relapse of multiple sclerosis in adults, relapse of multiple sclerosis in children, Guillain-Barré syndrome, chronic inflammatory disseminated polyneuropathy, opsoclonus myoclonus syndrome, or Bell’s palsy (*133*). ACIP considers Guillain-Barré syndrome occurring <6 weeks after receipt of a tetanus toxoid–containing vaccine a precaution for subsequent administration of tetanus toxoid–containing vaccines (*52*). Active surveillance data covering two million doses of Tdap administered to both adolescent and adult populations failed to demonstrate an association between receipt of a tetanus toxoid–containing vaccine and onset of Guillain-Barré syndrome within six weeks following vaccination (*164*,*165*,*217*).

##### Pregnant Women

Tetanus toxoid–containing vaccines are safe in pregnant women. No evidence exists to indicate that tetanus and diphtheria toxoids administered during pregnancy are teratogenic (*54*). Field trials of tetanus toxoid in pregnant women have shown high efficacy (80%–100%) in preventing maternal and neonatal tetanus (*50*,*218*–*222*).

##### Arthritis

Although the causal relation between vaccination with tetanus toxoid and arthritis is biologically plausible, the evidence of a possible association between receipt of tetanus vaccine and arthritis is limited (*132*). In 1994, on the basis of case reports, case series and uncontrolled observational studies, IOM concluded that the evidence was insufficient to demonstrate a causal link between receipt of tetanus toxoid and arthritis (*132*). In a second review of the evidence, IOM reached a similar conclusion on the basis of several case reports and two case-control studies (*133*). The first case-control study found an increased risk for psoriatic arthritis after tetanus toxoid vaccination (odds ratio [OR]: 1.91; 95% CI = 1.0%–3.7%) (*223*). In the second study, the investigators concluded that tetanus toxoid or diphtheria vaccination did not increase the risk for rheumatoid arthritis (*224*). Both studies had serious limitations and low precision (*133*).

A more recent study in a large health maintenance organization assessed the risk for rheumatoid arthritis after tetanus, influenza, and hepatitis B vaccination, using a cohort and case-control design to determine risk at different intervals postvaccination (*225*). This study did not identify a significantly increased risk for rheumatoid arthritis associated with tetanus vaccine for any interval assessed (*225*).

#### Diphtheria Toxoid Safety

Reactogenicity with vaccines containing diphtheria toxoid is common. All available pertussis-containing vaccines include diphtheria toxoid, and different forms of diphtheria toxoid are used as carrier proteins in certain conjugate vaccines (MenACWY-D [Menactra, Sanofi Pasteur], MenACWY-CRM [Menveo, GlaxoSmithKline, Rixensart, Belgium], 13-valent pneumococcal polysaccharide-protein conjugate vaccine [PCV13, Prevnar13, Wyeth Pharmaceuticals Inc., Collegeville, Pennsylvania, a subsidiary of Pfizer Inc., New York, New York]). The frequency of reported adverse events from diphtheria toxoid–containing vaccines varies by vaccine formulation, dose of diphtheria toxoid, prior vaccination history, and prevaccination antidiphtheria toxoid antibody levels. Although local injection-site reactions are common, only a small proportion of these are clinically significant (*226*). Administration of diphtheria toxoid has not been associated with anaphylaxis.

Because diphtheria toxoid is not administered as a monovalent diphtheria toxoid vaccine, it is difficult to characterize reactogenicity to diphtheria toxoid alone. However, in a study of 180 persons comparing the reactogenicity of DTaP (diphtheria toxoid ≥10 IU) with that of Td (diphtheria toxoid ≥2 IU) and of monovalent diphtheria toxoid (diphtheria toxoid ≥2 IU), the proportion of vaccinees with local reactions (e.g., erythema, induration, warmth, and tenderness) was generally lower among recipients of the monovalent diphtheria toxoid than was observed in the other two groups (*227*). There was no consistent pattern of increased reactogenicity among recipients of DTaP compared with Td (*227*). In addition, data from several controlled studies suggest that fever and local reactions are more common after administration of Td than after TT vaccine (*190*,*228*,*229*). In general, the frequencies of reported common systemic signs and symptoms in infants (i.e., temperature of ≥38º C [≥100.4º F], crying for ≥1 hour, irritability, drowsiness, loss of appetite, and vomiting) and local reactions (i.e., redness, swelling, and tenderness) after vaccination with DT or DTaP were comparable (*230*).

##### Pregnant Women

Diphtheria toxoid–containing vaccines are safe in pregnant women. No evidence exists to indicate that tetanus and diphtheria toxoids administered during pregnancy are teratogenic (*54*). Randomized trials of diphtheria toxoid conducted among pregnant women in the 1940s demonstrated efficient transplacental transfer of maternal antidiphtheria antibodies and protection of infants against diphtheria (*231*–*234*).

#### Simultaneous Administration of Tdap with Other Vaccines

Pre- and postlicensure studies in adolescents and adults have evaluated and support the safety and immunogenicity of Tdap when administered simultaneously or sequentially with one or two other vaccines (e.g., MenACWY, HepB, and human papillomavirus [HPV] and trivalent inactivated influenza vaccines) (*141*,*167*,*235*–*242*). In most of these studies, no differences were observed in the safety profiles when Tdap was administered simultaneously or sequentially with another vaccine. However, the rates of erythema and swelling at the Tdap injection site were higher when it was co-administered with HepB vaccine (*141*). Swollen or sore joints were reported in both the simultaneous (22.5%) and sequential (17.9%) vaccination groups, with most joint complaints being mild in intensity and lasting a mean duration of 1.8 days (*141*).

Tdap was immunogenic when administered simultaneously with other vaccines. The proportions of subjects achieving protective levels of antibodies against diphtheria and tetanus were similar in the simultaneously vaccinated group compared with those vaccinated sequentially (*235*–*242*). Immune responses to pertussis antigens were similar when Tdap was administered simultaneously or sequentially with MenACWY-D, HPV (bivalent or quadrivalent) or HepB vaccines, although the immune responses to pertussis antigens with MenACWY-CRM (Menveo, Novartis) and trivalent inactivated influenza vaccines were lower after simultaneous administration (*141*,*235*–*241*). When Tdap was administered simultaneously with MenACWY-CRM (Menveo, Novartis), the immune responses to two of the three pertussis antigens were lower, but the clinical relevance of this finding, if any, is not clear (*237*). When Tdap and trivalent inactivated influenza vaccines were administered to adults aged 19–64 years, the immune responses to the pertussis antigens were lower but noninferior in the simultaneously vaccinated group compared with the immue responses to the pertussis antigens in those vaccinated sequentially, with the exception of Adacel’s PRN and Boostrix’s FHA and PRN (*235*,*236*). The clinical significance, if any, of not meeting noninferiority criteria for these antigens is unclear (*235*). For adults aged ≥65 years, compared with separate administration of Boostrix and a trivalent inactivated influenza vaccine, simultaneous administration of these two vaccines also was safe and immunogenic (*242*).

#### Cost-Effectiveness Analyses

As part of the consideration of recommendations for use of Tdap in specific populations, cost-effectiveness analyses were conducted for specific vaccination strategies and target populations. These were considered for adults aged ≥65 years and women during pregnancy.

##### Vaccinating Adults Aged ≥65 Years

Two cost-effectiveness analyses of the epidemiologic and economic impact of substituting a single dose of Tdap for one decennial Td booster in healthy persons aged ≥65 years were reviewed by ACIP (CDC, unpublished data, 2012) (*243*). Both models were developed to assess the epidemiologic and economic impact of Tdap vaccination in adults aged ≥65 years, and both demonstrated that a dose of Tdap administered to older adults resulted in a modest decrease in the number of cases of pertussis and other outcomes (e.g., outpatient visits, hospitalizations, and deaths) (CDC, unpublished data, 2012) (*243*). From the two models with similar incidence (100–104 cases per 100,000 population), the cost per quality adjusted life-year saved ranged from $30,946 to $62,716 and the cost per case averted ranged from $1,966 to $3,263 (CDC, unpublished data, 2012) (*243*). Model results were most sensitive to the incidence of pertussis; however, sensitivity analyses showed that, even assuming a range of estimates of pertussis underreporting, Tdap vaccination compared with no Tdap vaccination might be cost-effective in this population. Reassured by the concordance between the two cost-effectiveness models, ACIP’s conclusion was that the cost per case averted and the cost per quality-adjusted life-year saved were modest (*10*).

##### Maternal Tdap Vaccination and Cocooning

A decision and cost-effectiveness model was developed to assess the likely impact and cost-effectiveness of Tdap vaccination administered during pregnancy versus postpartum with or without cocooning. The model showed that Tdap vaccination during pregnancy could reduce annual infant pertussis incidence more than postpartum vaccination, reducing cases by 33% versus 20%, hospitalizations by 38% versus 19%, and deaths by 49% versus 16%. The cost per quality adjusted life-year saved for pregnancy vaccination was $414,523 compared with postpartum vaccination, which was $1,172,825. The two primary drivers of the reduction in infant pertussis were earlier indirect protection from the mother by vaccinating before the infant’s birth and the provision of passive immunity to the infant through transplacental transfer of maternal antibodies. Sensitivity analyses under robust conditions, including reduced Tdap vaccine effectiveness, did not alter the relative benefits of vaccination during pregnancy (*244*).

## Strategy for Pertussis, Tetanus, and Diphtheria Control

### Routine DTaP, Tdap, and Td Vaccination

In the United States, reported tetanus and diphtheria cases are rare. Although vaccine coverage is high among infants, children, and adolescents, serologic and survey data indicate that adults are undervaccinated against tetanus and diphtheria and that coverage declines with increasing age (*36*,*51*). Maintaining seroprotection against tetanus and diphtheria through adherence to the ACIP-recommended schedule of booster doses of vaccine is important for adults of all ages.

In contrast to tetanus and diphtheria, the incidence of reported pertussis in the United States has been increasing despite high infant and childhood coverage with DTaP vaccines and increasing Tdap coverage among adolescents (*245*). Although vaccine-induced protection provided by acellular pertussis vaccines wanes over time, vaccination remains the best protection available against pertussis. ACIP recognizes that not all cases of pertussis can be prevented. However, sustaining vaccine coverage in young children (DTaP) and adolescents (Tdap) with the available licensed vaccines and achieving high Tdap coverage among adults, especially pregnant women, presents the best available means of preventing pertussis.

#### Preventing Pertussis in Young Infants Through Maternal Tdap Vaccination

Because young infants continue to be at greatest risk for hospitalization and death due to pertussis, ACIP has made efforts to optimize the vaccination program strategies for preventing pertussis in those too young to be vaccinated. Very young infants are dependent in part on passively acquired maternal antibodies, which are thought to protect infants from infection and to modify the severity of diverse infectious diseases in infants for varying periods of time (*246*,*247*). Before the ACIP recommendation to vaccinate pregnant women, several studies provided evidence supporting the existence of efficient transplacental transfer of pertussis antibodies (*181*,*248*,*249*). These studies indicated that newborn infants whose mothers received Tdap before or during pregnancy had higher concentrations of pertussis antibodies at birth compared with those of unvaccinated mothers (*181*,*248*,*249*).

The strategy of preventing pertussis in newborns through the vaccination of women with Tdap during pregnancy from 27 through 36 weeks’ gestation is 80%–91% effective (CDC, unpublished data, 2016) (*250*–*253*). One study found that, among infants infected with pertussis, those born to mothers vaccinated with Tdap during pregnancy had less severe pertussis than those born to unvaccinated mothers; maternal vaccination was 58% effective in preventing hospitalization among infants infected with pertussis (*254*).

##### Vaccinating From 27 Through 36 Weeks’ Gestation

Tdap may be administered any time during pregnancy, but vaccination during the third trimester likely provides the highest concentration of maternal antibodies to be transferred closer to birth (*247*). Substantial active transport of maternal immunoglobulin G does not take place before 30 weeks of gestation (*255*). After receipt of Tdap, a minimum of 2 weeks is required to mount a maximal immune response to the vaccine antigens (*256*,*257*). One study noted that, after receipt of Tdap, maternal antibodies waned quickly; pregnant women who received Tdap during the first or second trimester had low levels of antibodies at term, suggesting that Tdap might need to be administered later in pregnancy to have high levels of antibodies for transfer to infants (*247*). Therefore, to optimize the concentration of vaccine-induced antipertussis antibodies transported from mother to infant, ACIP concluded in 2012 that pregnant women should be vaccinated with Tdap during the third trimester, preferably from 27 through 36 weeks’ gestation (*6*).

New data available since 2012 suggest that vaccinating earlier in the 27 through 36-week time period will maximize passive antibody transfer to the infant (C. Mary Healy, Baylor College of Medicine, unpublished data, 2016) (*258*–*260*); however, it is unclear how this translates to effectiveness in preventing infant pertussis. Three studies have shown that, among infants whose mothers received Tdap during the 27 through 36 weeks’ gestational time period, antipertussis antibody concentrations were significantly higher in cord blood of infants whose mothers received Tdap “earlier” (e.g., 27 through 32 weeks’ gestation) compared with those who received Tdap “later” (e.g., after 32 weeks’ gestation) (C. Mary Healy, Baylor College of Medicine, unpublished data, 2016) (*259*,*260*). A fourth study indicated that those who received Tdap as early as 22 through 26 weeks’ gestation developed similar levels of antibody to those vaccinated 27 through 36 weeks’ gestation (*258*). These studies support the observation that vaccinating earlier within the 27 through 36 week period, or even slightly before 27 weeks, might optimize the production and transfer of maternal antibodies to infants.

Assuring a sufficient amount of time between a pregnant woman’s receipt of Tdap and her infant’s birth to allow for the maximum production and transfer of maternal antibodies is important and might be achieved by vaccinating at a gestational age earlier than the current guidance of 27 through 36 weeks. However, ACIP is cautious not to equate higher newborn maternal antibody concentrations, which might be achieved through earlier maternal Tdap vaccination, with similar or better effectiveness at preventing pertussis during infancy. Furthermore, it is unclear whether maternal vaccination earlier in pregnancy would result in the development and transfer of maternal antibodies at concentrations that would persist at protective levels until the infant’s first DTaP dose. Lacking effectiveness data on vaccination before 27 weeks’ gestation, ACIP concluded that vaccinating earlier in the 27 through 36 week period will maximize passive antibody transfer to the infant (*261*).

##### Vaccinating During Each Pregnancy

Studies of the persistence of antipertussis antibodies following a dose of Tdap show substantial decay in antibody levels after one year in healthy, nonpregnant adults (*186*,*262*,*263*); antibody kinetics in pregnant women are likely to be similar. With regard to maternal antipertussis antibody concentrations in infants born to women who received Tdap within the preceding 2 years, results from one study indicated that antipertussis antibody concentrations waned quickly in pregnant women vaccinated before pregnancy and were unlikely to be high enough to provide passive protection to infants (*247*). Because antibody levels wane substantially during the first year following vaccination, ACIP concluded that a single dose of Tdap during a pregnancy would be insufficient to provide protection for subsequent pregnancies (*6*).

##### Interference with Infant Immune Response to Primary Vaccination

The presence of maternally derived transplacental antipertussis antibodies might interfere with an infant’s response to subsequent active vaccination with recommended DTaP vaccines potentially putting an infant at risk for disease later in infancy. In the United Kingdom, infants born to mothers who received Tdap-IPV during pregnancy had lower PT, FHA, and FIM antibodies when measured 3–6 weeks after the third dose of a 2-3-4 month schedule, compared with infants born to unvaccinated mothers (*264*). Antibodies to diphtheria also were lower, as were antibodies to some CRM-conjugated pneumococcal antigens when vaccinated with PCV13 on a 2–4-month schedule. In contrast, antitetanus antibody and anti-Hib antibody responses were enhanced. In the United States and Canada, pertussis antibody levels were modestly diminished (7.2%–48.3%) following the third dose of a 2-4-6 month DTaP schedule in infants whose mothers received Tdap during pregnancy, compared with infants whose mothers were not vaccinated during pregnancy; however, after the fourth dose of DTaP, pertussis antibody levels were comparable in the two groups of infants (S. A. Halperin, Dalhousie University, unpublished data, 2011) (*192*,*249*). Because correlates of protection are not well defined for pertussis, the clinical importance of lower infant immune responses following receipt of DTaP is unclear. However, any interference with infant immune responses is likely to be short-lived because circulating maternal antibodies decline rapidly (*265*). Although it is not known what level of maternal antibody is protective against infant pertussis, ACIP concluded that the potential benefit of protection from maternal antibodies in newborn infants outweighs the potential risk for shifting pertussis disease burden to later in infancy and emphasized the importance of timely receipt of the fourth DTaP dose (*9*).

##### “Cocooning”

ACIP recommends Tdap vaccination for women during pregnancy to prevent infant pertussis. Before this recommendation was developed to vaccinate pregnant women with Tdap, the primary strategy to prevent infant pertussis was Tdap vaccination of close contacts of infants, on the assumption that vaccination with Tdap would reduce the risk for pertussis exposure and transmission to infants, a strategy referred to as “cocooning” (*5*).

Cocooning programs had limited success and have been confronted with substantial logistical and financial challenges to implementation and program sustainability (*43*,*266*–*268*). Programs achieved moderate Tdap coverage among postpartum mothers, but had less success vaccinating other family members (*268*,*269*). The evidence on the effectiveness and impact of cocooning in preventing transmission of pertussis to infants is inconclusive (*153*,*154*,*270*). Recent epidemiologic and animal model evidence suggests that Tdap vaccination does not prevent transmission and therefore does not afford indirect protection of close contacts against pertussis (*155*,*156*,*159*). However, persons who are up to date with pertussis vaccines and who become infected generally have a milder infection compared with those who have not been vaccinated, which might make them less efficient in transmitting pertussis to others (*160*).

When recommendations for Tdap vaccination were made in 2005, mothers were identified as the primary source of pertussis in infants (*271*,*272*). This appears to have shifted, with siblings now identified as the primary source of pertussis infection for infants (*40*,*42*,*43*). This shift in the source of infant pertussis, along with recent evidence suggesting that Tdap vaccination does not prevent transmission of *B. pertussis*, underscores the importance of providing newborns with maternal antipertussis antibodies through Tdap vaccination of women during pregnancy (*155*,*156*,*159*). A single dose of Tdap is recommended for all persons aged ≥11 years who have not previously received a dose; having a pregnancy in a household presents an opportunity to review the Tdap vaccination status of close contacts to ensure that they are up to date.

#### Preventing Pertussis in Health Care Personnel

Health care personnel in the United States are not known to have higher risk for diphtheria or tetanus compared with the general population. However, for pertussis, occupational exposures occur in health care settings, and nosocomial spread of pertussis in various health care settings has been documented (*273*–*288*). In these settings, the index case might occur in a health care provider, patient, or hospital visitor (*273*–*288*). Although the frequency and intensity of patient exposure might lead to infection of health care personnel with subsequent transmission to other patients, the risk for and burden of pertussis in health care personnel are difficult to quantify. The few population-based estimates of the risk for pertussis among health care personnel in the United States suggest the risk for pertussis among health care personnel is comparable to the risk among the general population of adolescents and adults (*289*–*291*).

Since 2005, ACIP has recommended that health care personnel receive a single dose of Tdap to protect them against pertussis and possibly reduce transmission to patients, co-workers, household members, and persons in the community. Hospital-based Tdap coverage rates among health care personnel might depend on the type of Tdap vaccination program an institution employs; reported coverage ranges from 30% (campaign) to 100% (hospital mandate) (*292*,*293*). Nationally, Tdap coverage among health care personnel is 42.1% (*36*).

Previous models assessing the likely benefits and costs of vaccinating health care personnel with Tdap to prevent nosocomial pertussis outbreaks indicated that vaccination of health care personnel substantially reduced the risk for hospital-based outbreaks and was cost-saving (*294*,*295*). However, model inputs included estimates of Tdap vaccine efficacy against pertussis higher than current estimates, and assumed vaccination would decrease pertussis transmission and thereby prevent secondary cases. Current data do not support the assumption that Tdap vaccination would prevent transmission.

##### Management of Health Care Personnel Exposed to Pertussis

Depending on the approach used, management of pertussis exposures in health care settings can be complicated, time-consuming, and costly. Exposed health care personnel with cough illness must be evaluated and might require diagnostic testing, administration of prophylactic antimicrobial agents, and possible exclusion from work. Several studies have shown that the costs of investigating pertussis outbreaks in the health care setting and implementing control measures are substantial. The cost of managing pertussis exposures in the health care setting over a 12-month period ranged from $84,000 to $98,000 (*284*,*294*). The associated costs of dealing with hospital-based pertussis outbreaks ranged from $74,000 to $263,000 (*285*,*288*). Since the promulgation of the 2005 Tdap recommendations for health care personnel, only one study has tried to determine whether it is necessary to give Tdap-vaccinated health care personnel postexposure antimicrobial prophylaxis, but the results were inconclusive due to the low risk for disease during the study period (*296*).

##### Guidance on Postexposure Prophylaxis for Health Care Personnel

Tdap vaccination status does not change the approach to evaluating postexposure prophylaxis when health care personnel are exposed to pertussis. Postexposure prophylaxis is recommended for health care personnel in contact with persons at risk for severe disease (e.g., hospitalized neonates, newborn infants, and patients with chronic respiratory conditions). Other health care personnel can either receive postexposure prophylaxis or be carefully monitored for 21 days after pertussis exposure. Health care personnel should be treated with antibiotics at the onset of signs and symptoms of pertussis, and excluded from work for the first 5 days while receiving appropriate antibiotics. Recommended antimicrobial agents for postexposure prophylaxis among health care personnel exposed to pertussis include azithromycin, clarithromycin, erythromycin, and trimethoprim-sulfamethoxazole (TMP-SMX). Guidance on postexposure prophylaxis of pertussis is available at https://www.cdc.gov/mmwr/PDF/rr/rr5414.pdf.

#### No Additional Doses of Tdap for the General Population

With the exception of pregnant women, only a single booster dose of Tdap is recommended for persons aged ≥11 years. Both available Tdap products are approved for use as a single booster dose (*141*,*142*). Tdap provides protection against tetanus, diphtheria, and pertussis, but protection from pertussis infection begins to decline within 2 to 4 years after receipt of Tdap (*29*,*149*,*150*).

Clinical trials support that a second dose of Tdap is safe and immunogenic at a 5- or 10-year interval (*184*–*188*). Immunogenicity studies show that diphtheria and tetanus antibody levels persisted for five to 10 years after receipt of Tdap (*186*,*188*,*262*,*263*,*297*–*301*). However, antipertussis antibodies decline rapidly after the first year, suggesting that protection wanes, which would likely limit the impact of a second dose of Tdap on the overall burden of pertussis in the United States (*186*,*188*,*262*,*263*,*297*–*301*). Antibody decay and, therefore, clinical protection following additional doses of Tdap are likely similar to what is observed following the first dose of Tdap.

ACIP recognizes the increasing burden of pertussis in the United States and the need for an effective strategy to reduce this burden. A decision analysis model evaluating the epidemiologic and economic impact of a routine program of additional doses of Tdap administered at either a 5- or 10-year interval to persons who received their first Tdap at age 11 years suggested that the reduction in pertussis disease burden attributable to the routine use of a second dose of Tdap would be limited (*302*). In the model, the proportion of cases preventable compared with the recommendation, ranged from 3% to 5% (*302*). ACIP concluded that the data do not support a general recommendation for a routine second dose of Tdap, and that the public health impact of routinely recommending a second dose of Tdap would be limited (*303*).

## Rationale for Recommendations for Use of Pertussis, Tetanus, and Diphtheria Vaccines

Before the availability of vaccines, pertussis, tetanus, and diphtheria were common diseases and caused severe morbidity and mortality. As a result of the routine DTP/DTaP childhood vaccination program and decennial booster doses of tetanus-toxoid containing vaccines for adolescents and adults, the number of cases of all three diseases has declined markedly; cases of tetanus and diphtheria are rare in the United States. For pertussis, the number of reported cases declined dramatically following introduction of universal childhood pertussis vaccination (*3*). However, even with sustained high vaccine coverage, the incidence of reported pertussis began increasing in the 1980s.

In 1997, ACIP recommended that DTaP replace all DTP doses; since then, no changes have been made to these childhood vaccine recommendations (*3*). In 2005, to address the increase in the incidence of pertussis among adolescents and adults, ACIP recommended a single dose of Tdap for persons aged 11–64 years as a booster immunization against tetanus, diphtheria, and pertussis, with the intention to provide routine vaccination at ages 11–12 years (*4*,*5*). Additionally, special populations, including health care personnel and close contacts of infants, were recommended to receive a dose of Tdap.

Since the promulgation of the ACIP Tdap recommendations in 2005, several studies have identified barriers to and programmatic gaps in the implementation of the recommendations for adolescents and adults. Barriers to implementation included confusion around guidance language concerning the timing of administration of Tdap after the last tetanus toxoid–containing vaccine dose and obstacles to vaccinating health care personnel. Programmatic gaps at the time of the 2005 recommendations included lack of a Tdap vaccine licensed for children aged 7–10 years and for adults aged ≥65 years. In 2011 and 2012, ACIP recommended a dose of Tdap for these age groups and clarified the language concerning the timing of Tdap vaccination and the vaccination of health care personnel (*8*,*10*,*304*).

Compared with older children and adults, infants aged <12 months have substantially higher rates of pertussis (Figure 2) and the largest burden of pertussis-related deaths. The majority of pertussis-related hospitalizations and deaths occur in infants aged ≤2 months who are too young to be vaccinated. The desire to protect the youngest infants from pertussis morbidity and mortality prompted ACIP in 2011 to recommend a dose of Tdap be administered to women during pregnancy, but only for pregnant women who had never received Tdap (*9*). Because antibody levels wane substantially after vaccination, ACIP concluded that a single dose of Tdap during one pregnancy would not provide protection for infants who were the product of subsequent pregnancies. In 2012, the recommendation was revised; ACIP recommends the use of Tdap during each pregnancy (*6*).

## Recommendations for Vaccination for Pertussis, Tetanus, and Diphtheria

All persons are recommended to receive routine pertussis, tetanus, and diphtheria vaccination. Vaccine type, product, number of doses and booster dose recommendations are based on age and pregnancy status (Tables 4 and 5). Recommendations for off-label use of Tdap vaccines were made after thorough review of available data on the risks for and benefits of Tdap vaccination, and include the following persons: pregnant women, children aged 7–10 years, and persons aged ≥65 years (for one Tdap product) (*6*,*8*–*10*). At the time these recommendations were made, ACIP determined that although data were limited, the benefits of off-label Tdap vaccination in preventing pertussis and decreasing pertussis-related morbidity and mortality outweigh the risks of an adverse event.

### General Recommendations

#### Persons Aged 2 Months–6 Years

The routine pertussis, tetanus, and diphtheria vaccination schedule for persons aged 2 months–6 years is comprised of five doses of vaccine containing diphtheria and tetanus toxoids, and pertussis antigens (DTaP), administered at ages 2, 4, 6, 15–18 months and 4–6 years.

- Three (primary) doses should be administered at ages 2, 4, and 6 months.
- The fourth (first booster) dose should be administered to children aged 15–18 months to maintain adequate immunity during preschool years.
- The fifth (second booster) dose should be administered to children aged 4–6 years to confer continued protection against disease during the early years of schooling.

**Guidance for Use**

The first DTaP dose can be administered as early as age 6 weeks.

The fourth DTaP dose should be administered at least 6 months after the third DTaP dose and should not be administered to a child aged <12 months.

A fifth DTaP dose is not necessary if the fourth DTaP dose in the series is administered at age ≥4 years.

#### Persons Aged 11–18 Years

Persons aged 11–18 years should receive a single dose of Tdap, preferably at a preventive care visit at ages 11–12 years. To ensure continued protection against tetanus and diphtheria, booster doses of Td should be administered every 10 years throughout life.

#### Persons Aged ≥19 Years

Persons aged ≥19 years who previously have not received a dose of Tdap should receive a single dose of Tdap in place of a decennial Td booster dose. The dose of Tdap, when indicated, should not be delayed and should be administered regardless of interval since the last tetanus or diphtheria toxoid–containing vaccine. To ensure continued protection against tetanus and diphtheria, booster doses of Td should be administered every 10 years throughout life.

#### Pregnant Women

ACIP recommends that providers of prenatal care implement a Tdap immunization program for all pregnant women. Health care personnel should administer a dose of Tdap during each pregnancy, irrespective of the patient's prior history of receiving the vaccine.

**Guidance for Use**

Tdap should be administered between 27 and 36 weeks’ gestation, although it may be administered at any time during pregnancy. Available data suggest that vaccinating earlier in the 27–36 week time period will maximize passive antibody transfer to the infant. 

Tdap may be simultaneously administered with an inactivated influenza vaccine to pregnant women.

If a woman did not receive Tdap during her current pregnancy and did not receive a prior dose of Tdap ever (i.e., during adolescence, adulthood, or a previous pregnancy), then Tdap should be administered immediately postpartum. If a woman did not receive Tdap during her current pregnancy but did receive a prior dose of Tdap, then she should not receive a dose of Tdap postpartum.

### Vaccination of Special Populations

#### Persons Aged 2 Months–6 Years

**Contraindications to pertussis vaccination**. For children aged <7 years with a contraindication to pertussis vaccination (Table 2), DT should be used instead of DTaP to complete an age appropriate series. Previously unvaccinated children who receive their first DT dose at age <12 months should receive a total of 4 doses of DT, the first 3 doses at 4- to 8-week intervals and the fourth dose 6 to 12 months later (similar to the recommended DTaP schedule).

Unvaccinated children aged ≥12 months for whom pertussis vaccine is contraindicated should receive 2 doses of DT 4 to 8 weeks apart, followed by a third dose 6 to 12 months later to complete the primary series. Children who have already received 1 or 2 doses of DT or DTaP after their first birthday and for whom further pertussis vaccine is contraindicated should receive a total of 3 doses of a preparation containing diphtheria and tetanus toxoids appropriate for age, with the third dose administered 6 to 12 months after the second dose.

Children aged 4–6 years who complete a primary series of DT before their fourth birthday should receive a fifth dose of DT by the time of school entry to confer continued protection against disease.

**Personal history of seizures.** Among infants and children with a history of previous seizures, it is prudent to delay pertussis vaccination until the child’s neurologic status has been assessed. Infants and children with a stable neurologic condition, including well-controlled seizures, may be vaccinated with DTaP. Infants with evolving neurologic conditions should not be vaccinated until a treatment regimen has been established and the condition has stabilized. A family history of seizures is not a contradiction to pertussis vaccination.

#### Persons Aged ≥11 Years

**Close contacts of infants.** Persons aged ≥11 years who have or anticipate having close contact with an infant aged ≤12 months (e.g., parents, siblings, grandparents, child care providers, and health care providers) and who have never received Tdap should receive a dose of Tdap. Ideally, these persons should receive Tdap at least 2 weeks prior to contact with the infant to allow for an immune response to pertussis vaccine antigens.

**Health care personnel.** All health care personnel should receive a single dose of Tdap as soon as feasible if they have not previously received Tdap. After receipt of 1 dose of Tdap, health care personnel should receive routine Td booster immunizations according to the recommended schedule.

In-patient and out-patient care facilities should consider approaches that maximize vaccination rates of health care personnel (e.g., education about the benefits of vaccination, convenient access, provision of Tdap at no charge).

### Persons with Incomplete or Unknown Vaccine History

#### Persons Aged 2 Months–6 Years

For persons aged <7 years not fully immunized with DTaP vaccine, the catch-up schedule and minimum intervals between doses are available at https://www.cdc.gov/vaccines/schedules/hcp/child-adolescent.html. The vaccine series does not need to be restarted regardless of the time that has elapsed between doses for those with incomplete DTaP vaccine history.

Because of concern about adverse reactions, the total number of doses of vaccines containing diphtheria and tetanus toxoids (e.g., DTaP, DT, and DTP) received should not exceed 6 doses before the seventh birthday. Only documented doses count toward the maximum of 6 doses.

#### Persons Aged 7–18 Years

Persons aged 7–18 years not fully immunized with DTaP vaccine should receive a single dose of Tdap as one (preferably the first) dose of the catch-up series; if additional doses are needed, use Td vaccine. The vaccine series does not need to be restarted, regardless of the time that has elapsed between doses for those with incomplete DTaP vaccine history. The catch-up schedule and minimum intervals between doses are available at https://www.cdc.gov/vaccines/schedules/hcp/child-adolescent.html.

For persons aged 7–10 years who receive a dose of Tdap as part of the catch-up series, an adolescent Tdap vaccine dose should be administered at age 11–12 years.

#### Persons Aged >18 Years

Persons aged >18 years who have never been vaccinated against pertussis, tetanus, or diphtheria should receive a series of three vaccinations containing tetanus and diphtheria toxoids, which includes 1 dose of Tdap. The preferred schedule is a single dose of Tdap, followed by a dose of Td at least 4 weeks after Tdap and another dose of Td 6 to 12 months later. However, the single dose of Tdap can substitute for any of the Td doses in the 3-dose primary series.

Persons aged >18 years who are not fully immunized against tetanus and diphtheria should receive 1 dose of Tdap (preferably the first) in the catch-up series; if additional tetanus toxoid–containing doses are needed, use Td vaccine. Alternatively, in situations in which a person aged >18 years probably received vaccination against tetanus and diphtheria but cannot produce documentation, vaccine providers may consider serologic testing for antibodies to tetanus and diphtheria toxin to avoid unnecessary vaccination. If tetanus and diphtheria antitoxin levels are each >0.01 IU/mL, previous vaccination with tetanus and diphtheria toxoid vaccine is presumed, and a single dose of Tdap is indicated.

### Prevention of Obstetric and Neonatal Tetanus

Pregnant women who have completed the childhood immunization schedule and were last vaccinated more than ten years previously should receive a booster dose of tetanus toxoid–containing vaccine to prevent neonatal tetanus. The risk of neonatal tetanus is minimal if a previously unimmunized woman has received at least 2 properly spaced doses of tetanus toxoid–containing vaccine during pregnancy; one of the doses administered during pregnancy should be Tdap, administered according to the current guidance. She should complete the 3-dose primary series at the recommended intervals.

### Tetanus Prophylaxis for Wound Management

ACIP has recommended administering tetanus toxoid–containing vaccine and tetanus immune globulin (TIG) when indicated as part of standard wound management to prevent tetanus (Table 6). A tetanus toxoid–containing vaccine is indicated as part of wound management if more than five years has passed since the last tetanus toxoid–containing vaccine dose. If a tetanus toxoid–containing vaccine is indicated for persons aged ≥11 years, Tdap is preferred for persons who have not previously received Tdap or whose Tdap history is unknown. If a tetanus toxoid–containing vaccine is indicated for a pregnant woman, Tdap should be used. For nonpregnant persons with documentation of previous vaccination with Tdap, Td should be used if a tetanus toxoid–containing vaccine is indicated. If a tetanus toxoid–containing vaccine is indicated and Td is unavailable, Tdap may be administered.

**TABLE 6:** Guide to tetanus prophylaxis in routine wound management

No. doses of adsorbed tetanus toxoid–containing vaccines	Clean and minor wound		All other wounds*	
	DTaP, Tdap, or Td^†§^	TIG	DTaP, Tdap, or Td^†^	TIG^§^
Unknown or <3	Yes	No	Yes	Yes
≥3	No^¶^	No	No**	No

**Abbreviations**: DTaP = diphtheria and tetanus toxoids and acellular pertussis vaccine; Tdap = tetanus toxoid, reduced diphtheria toxoid, and acellular pertussis; Td = tetanus and diphtheria toxoids; TIG = tetanus immune globulin.

* Such as, but not limited to, wounds contaminated with dirt, feces, soil, and saliva; puncture wounds; avulsions; and wounds resulting from missiles, crushing, burns, and frostbite.

^†^ DTaP is recommended for children aged <7 years. Tdap is preferred to Td for persons aged ≥11 years who have not previously received Tdap. Persons aged ≥7 years who are not fully immunized against pertussis, tetanus or diphtheria should receive one dose of Tdap for wound management and as part of the catch-up series.

^§^ Persons with HIV infection or severe immunodeficiency who have contaminated wounds should also receive TIG, regardless of their history of tetanus immunization.

^¶^Yes, if >10 years since the last tetanus toxoid–containing vaccine dose.

** Yes, if ≥5 years since the last tetanus toxoid–containing vaccine dose.

Persons who have completed the 3-dose primary tetanus vaccination series and have received a tetanus toxoid–containing vaccine <5 years earlier are protected against tetanus and do not require a tetanus toxoid–containing vaccine or TIG as part of wound management. An attempt should be made to determine whether a patient has completed the 3-dose primary tetanus vaccination series. Persons with unknown or uncertain previous tetanus vaccination histories should be considered to have had no previous tetanus toxoid–containing vaccine. Persons who have not completed the primary series might require tetanus toxoid–containing vaccine and passive vaccination with TIG at the time of wound management (Table 6). When both TIG and a tetanus toxoid–containing vaccine are indicated, the products should be administered using separate syringes at different anatomical sites. Persons with human immunodeficiency virus (HIV) infection or severe immunodeficiency who have contaminated wounds should also receive TIG, regardless of their history of tetanus immunizations.

Persons with a history of an Arthus reaction following a previous dose of a tetanus toxoid–containing vaccine should not receive a tetanus toxoid–containing vaccine until >10 years after the most recent dose; this interval is recommended regardless of the wound condition (e.g., even if contaminated or severe). In all circumstances, the decision to administer TIG should be based on the primary vaccination history for tetanus (Table 6).

### Special Situations

#### Accelerated Schedule for Infants and Children Aged <7 Years

For an infant or child aged <7 years, circumstances such as travel, potential loss to follow-up or increased risk of exposure to pertussis might warrant an accelerated schedule to provide protection as early as possible. An accelerated schedule can be started as soon as the infant is aged 6 weeks, with the second and third DTaP doses administered no earlier than 4 weeks after each preceding dose. The fourth DTaP dose should not be administered before the infant is aged 12 months and should be separated from the third dose by at least 6 months. The fifth DTaP dose should not be administered before the child is aged 4 years. When considering an accelerated schedule, providers also should consider the timing of other recommended vaccines and well-child visits.

#### History of Pertussis

Persons who have a history of pertussis should receive a pertussis-containing vaccine (i.e., DTaP or Tdap) according to the routine recommendation. Although pertussis disease is likely to confer natural immunity against pertussis, the immune response might be suboptimal against subsequent pertussis disease and the duration of protection induced by an infection does not provide long-term immunity (*305*,*306*).

#### Persons Who Have Recovered from Tetanus or Diphtheria

Tetanus or diphtheria infection do not necessarily confer immunity against re-infection (*54*,*307*,*308*); therefore, active vaccination should be initiated at the time of recovery from the illness according to the schedule. Persons who have completed the primary tetanus vaccination series should receive a booster dose as soon as feasible during convalescence. Persons with unknown or uncertain previous tetanus vaccination histories should be considered to have had no previous tetanus toxoid–containing vaccine and should begin the 3-dose tetanus and diphtheria toxoids vaccination series.

### Vaccine Administration

A summary of dose schedules is provided (Tables 4 and 5).

All health care personnel administering vaccinations should be aware of the potential for syncope after vaccination, especially among adolescents and young adults, and should take appropriate measures to prevent potential injuries. Providers should strongly consider observing patients for 15 minutes after they are vaccinated. If syncope occurs, the vaccine recipient should be observed until symptoms resolve (*52*).

#### DTaP

Six vaccines are licensed for the pertussis, tetanus and diphtheria vaccination series (Table 4). The dose of DTaP is 0.5 mL, administered intramuscularly. The preferred intramuscular injection site for infants and children through age 2 years is the anterolateral aspect of the thigh (*52*). For children aged ≥3 years, the preferred site is the deltoid muscle (*52*). DTaP may be administered simultaneously with other vaccines at a different anatomical site.

**Interchangeable use of acellular pertussis vaccines.** Whenever feasible, the same DTaP product should be used for all doses of the vaccination series. Data are limited regarding the safety, immunogenicity, and efficacy of using DTaP vaccines from different manufacturers for successive doses of the primary or booster vaccination series. However, the vaccine provider might not know or have available the type of DTaP vaccine previously administered to a child; neither circumstance should present a barrier to administration of the vaccine. Any of the licensed DTaP vaccines may be used to complete the vaccination series.

**Minimum interval between third and fourth DTaP dose.** The recommended minimal interval between the third and fourth doses of DTaP is 6 months, and the minimum age for receipt of the fourth dose of DTaP is 12 months. However, a fourth DTaP dose is considered valid if administered at least 4 months after the third dose of DTaP and the child is aged ≥12 months.

#### DT

One vaccine is licensed for active immunization of children up to age seven years against diphtheria and tetanus in instances where the pertussis vaccine component is contraindicated or where the physician decides that pertussis vaccine is not to be administered (Table 4). The dose of DT is 0.5 mL, administered intramuscularly. The preferred intramuscular injection site for infants and children through age 2 years is the anterolateral aspect of the thigh (*52*). For children aged ≥3 years, the preferred site is the deltoid muscle (*52*).

#### Tdap

Two vaccines are licensed for the pertussis, tetanus and diphtheria vaccination booster dose for adolescents and adults (Table 5). The dose of Tdap is 0.5 mL, administered intramuscularly, preferably into the deltoid muscle (*52*). Tdap may be administered simultaneously with other vaccines at a different anatomical site.

**Interval between Td and Tdap.** ACIP recommends that for pertussis vaccination, when indicated, Tdap should be administered regardless of interval since the last tetanus or diphtheria toxoid–containing vaccine. ACIP concluded that, while longer intervals between Td and Tdap vaccination could decrease the occurrence of local reactions, the benefits of protection against pertussis outweigh the potential risk for adverse events. For persons aged ≥7 years with incomplete or unknown vaccine history, the interval between doses of tetanus toxoid–containing vaccines should follow the catch-up series schedule.

**Tdap products in adults aged ≥65 years.** Providers should not miss an opportunity to vaccinate persons aged ≥65 years with Tdap. When feasible, Boostrix (approved for use in persons aged ≥10 years) should be used for adults aged ≥65 years instead of Adacel (approved for use in persons aged 10–64 years); however, ACIP concluded that either vaccine administered to a person aged ≥65 years is immunogenic and would provide protection. A dose of either Tdap product is considered valid; therefore, providers may administer the Tdap vaccine they have available.

#### Td

For tetanus and diphtheria toxoids adsorbed vaccines, there are two licensed vaccines (Table 5). The dose of Td is 0.5 mL, administered intramuscularly, preferably into the deltoid muscle (*52*).

### Inadvertent Administration

#### DTaP

DTaP is not indicated for persons aged ≥7 years. If DTaP is administered inadvertently to a fully vaccinated child aged 7–10 years, this dose should be counted as the adolescent Tdap dose. If DTaP is administered inadvertently to an undervaccinated child aged 7–10 years, this dose should count as the Tdap dose of the catch-up series and the child should receive an adolescent booster dose of Tdap. If DTaP is administered inadvertently to a person aged ≥11 years, this dose should count as the Tdap dose, and the person should not receive an additional dose of Tdap.

#### Tdap

**Persons aged 2 months–6 years.** If Tdap is administered inadvertently instead of DTaP as any one of the first 3 doses of the tetanus-diphtheria-pertussis vaccination series, the Tdap dose should not be counted as valid, and a replacement dose of DTaP should be administered. The replacement dose of DTaP can be administered as soon as feasible at any interval after the inadvertent Tdap dose. The remaining doses of the DTaP series should be administered on the routine schedule, with at least a four-week interval between the replacement dose of DTaP and the next dose of DTaP. The adolescent Tdap dose should be administered as recommended when this child is aged 11–12 years.

If Tdap is administered inadvertently as the fourth or the fifth dose in the tetanus-diphtheria-pertussis vaccination series, the Tdap dose should be counted as valid and does not need to be repeated; the child who received Tdap as a fourth dose should complete the pediatric DTaP schedule. The adolescent Tdap dose should be administered as recommended when this child is aged 11–12 years.

**Children aged 7–10 years who are fully vaccinated.****^¶^** If Tdap is administered inadvertently, the Tdap dose should not be counted as valid. The adolescent Tdap dose should be administered as recommended when this child is aged 11–12 years.

### Additional Doses of Tdap for the General Population

Both Tdap products are licensed for use as a single dose for active booster immunization; Boostrix is approved for use in persons aged ≥10 years and Adacel is approved for use in persons aged 10–64 years. Tdap is not licensed for multiple administrations nor is it recommended for multiple administrations, with the exception of the recommendation that pregnant women receive a dose of Tdap during each pregnancy. If a dose of Tdap is administered to a person who has previously received Tdap, this dose should count as the next booster dose of tetanus toxoid–containing vaccine.

### Contraindications and Precautions

Providers should screen patients for contraindications and precautions to the vaccine before each dose of vaccine is administered (Table 2). A contraindication is a condition in a recipient that increases the risk for a serious adverse reaction. A vaccine should not be administered when a contraindication is present. In contrast, certain conditions are commonly misperceived as contraindications (i.e., are not valid reasons to defer vaccination) (Table 3). In general, vaccinations should be deferred when a precaution is present. However, a vaccination might be indicated in the presence of a precaution if the perceived benefit of protection from the vaccine outweighs the risk for an adverse reaction.

For DTaP vaccines, providers and parents should evaluate the risks and benefits of administering subsequent doses of a pertussis-containing vaccine. In circumstances in which the benefits of further pertussis vaccination outweigh the possible risks (e.g., during an outbreak of pertussis), DTaP vaccine should be administered for the subsequent doses.

### Reporting of Vaccine Adverse Events

Clinically significant and serious adverse events that arise after vaccination should be reported to VAERS at https://vaers.hhs.gov/reportevent.html. VAERS is a postmarketing safety surveillance program that collects information about adverse events (possible side effects) that occur after the administration of vaccines licensed for use in the United States.

Reports can be filed securely online, by mail, or by fax. A VAERS form can be downloaded from the VAERS website or requested by e-mail (info@vaers.org), telephone (800-822-7967), or fax (877-721-0366). Additional information on VAERS and vaccine safety is available at https://vaers.hhs.gov/about/index or by calling telephone 800-822-7967.

## National Vaccine Injury Compensation Program

The National Vaccine Injury Compensation Program (VICP), established by the National Childhood Vaccine Injury Act of 1986, as amended, provides a mechanism through which compensation can be paid on behalf of a person determined to have been injured or to have died as a result of receiving a vaccine covered by VICP. National Childhood Vaccine Injury Act requires health care providers to report any adverse events listed by the manufacturer as a contraindication to further vaccination or any adverse event listed in the VAERS Table of Reportable Events Following Vaccination that occurs within the specified time period after vaccination. The Vaccine Injury Table lists the vaccines covered by VICP and the injuries and conditions (including death) for which compensation might be paid. If the injury or condition is not included in the table or does not occur within the time period specified on the table, persons must prove that the vaccine caused the injury or condition. For a person to be eligible for compensation, the general filing deadlines for injuries require claims to be filed within 3 years after the first sign or symptom of the vaccine injury; for a death, claims must be filed within 2 years of the vaccine-related death and not more than 4 years after the start of the first sign or symptom of the vaccine-related injury from which the death occurred. When a new vaccine is covered by VICP or when a new injury/condition is added to the table, claims that do not meet the general filing deadlines must be filed within 2 years from the date the vaccine or injury/condition is added to the table for injuries or deaths that occurred up to 8 years before the table change. Persons who receive a VICP-covered vaccine might be eligible to file a claim. Additional information about VICP is available https://www.hrsa.gov/vaccinecompensation/index.html or by calling 800–338–2382.

## Safety Monitoring in Pregnant Women

Safety monitoring in pregnant women following Tdap administration includes enhanced monitoring in VAERS and utilization of the VSD to assess acute adverse events during pregnancy, adverse pregnancy outcomes affecting the mother, and birth outcomes.

Although not required by the FDA, pregnancy registries were established by Sanofi Pasteur and GSK during licensure of both Tdap vaccines to collect data on adverse events following inadvertent administration of Tdap vaccine during pregnancy. ACIP recommends administration of Tdap vaccine during each pregnancy. Neither Tdap product is contraindicated for use in pregnant women; lack of a specific “indication and usage” statement about use of the product in pregnant women in the product labeling does not preclude use of these vaccines during pregnancy. Both pharmaceutical companies continue to maintain a pregnancy registry. Health care providers may report Tdap vaccination during pregnancy, regardless of trimester, to the appropriate company’s pregnancy registry: Sanofi Pasteur (Adacel) telephone: 800-822-2463 and GSK (Boostrix) telephone: 888-452-9622.

## Future Directions

The United States has experienced substantial increases in the number of reported pertussis cases and changes in the epidemiologic features of pertussis since the early 1990s. The impact of switching from whole-cell pertussis vaccines (DTP) to acellular pertussis vaccines (DTaP) on the epidemiologic features of pertussis is still being investigated. Both DTaP and Tdap vaccines remain the most effective tools for preventing pertussis disease and are associated with fewer serious adverse events than DTP, but data thus far indicate that they do not provide long-term protection and might not prevent transmission.

Since the introduction of a single Tdap booster vaccine for adolescents and adults in 2005, changes to the recommendation were made in order to reduce barriers to Tdap uptake and coverage among adolescents and adults and to reduce the burden of pertussis in infants. High vaccine coverage in adolescents is being achieved and has met the *Healthy People 2020* target (80%), but attaining high coverage among adults remains a challenge (*35*). Despite challenges to vaccinating women during pregnancy, focused efforts to educate providers and pregnant women have resulted in gradual improvement in Tdap coverage. Ongoing efforts are needed to increase Tdap coverage in each pregnancy to optimize prevention of severe pertussis in young infants. The effects of the changes made to the Tdap recommendations need to be monitored and evaluated over time for their effectiveness and impact on pertussis, particularly in infants.

As the epidemiologic features of pertussis in the United States continue to evolve and more is understood about acellular pertussis vaccines, reassessment of immunization strategies might be required in the future. Looking ahead, the ability to improve the current prevention and control strategies is limited by gaps in the understanding of the immune response to acellular pertussis vaccines and pertussis infection, as well as the lack of accepted defined serologic or laboratory correlates of protection against pertussis. Given existing understanding of the durability of protection provided by the current pertussis vaccines, optimizing the current pertussis vaccination program and protecting infants, who are at highest risk for pertussis-related death, are immediate priorities.

## Footnotes

* A list of the members of the Work Group appears on page 43.

† Information available at http://wwwnc.cdc.gov/travel/page/yellowbook-home.

§ The case definition was a confirmed case of pertussis with ≥21 days of paroxysmal cough illness with culture or serologic confirmation of infection with *B. pertussis*.

¶ Fully vaccinated is defined as having received 5 valid doses of DTaP or 4 valid doses of DTaP if the fourth dose was administered on or after the fourth birthday.
